# Genome-wide DNA methylation profiles of colorectal tumors in Lynch syndrome and familial adenomatous polyposis

**DOI:** 10.1186/s13148-025-01940-x

**Published:** 2025-08-02

**Authors:** Satu Mäki-Nevala, Anni Kauppinen, Alisa Olkinuora, Aleksi Laiho, Petri Törönen, Laura Renkonen-Sinisalo, Anna Lepistö, Toni T. Seppälä, Jukka-Pekka Mecklin, Päivi Peltomäki

**Affiliations:** 1 Department of Medical and Clinical Genetics, Faculty of Medicine, University of Helsinki, Helsinki, Finland; 2 Organismal and Evolutionary Biology Research Program, Faculty of Biosciences, and Institute of Biotechnology, Helsinki Institute of Life Science (HiLIFE), University of Helsinki, Helsinki, Finland; 3 Department of Surgery, Helsinki University Hospital, Helsinki, Finland; 4 Applied Tumor Genomics, Research Programs Unit, University of Helsinki, Helsinki, Finland; 5 Department of Gastroenterology and Alimentary Tract Surgery, Tampere University Hospital and TAYS Cancer Centre, Tampere, Finland; 6 Department of Science, Well Being Services County of Central Finland, Jyväskylä, Finland; 7 Faculty of Sports and Health Sciences, University of Jyväskylä, Jyväskylä, Finland; 8 HUSLAB Laboratory of Genetics, HUS Diagnostic Center, Helsinki University Hospital, Helsinki, Finland

**Keywords:** Lynch syndrome, Familial adenomatous polyposis, Colorectal cancer, Colon adenoma, DNA methylation, Tumorigenesis

## Abstract

**Background:**

Lynch syndrome (LS) and familial adenomatous polyposis (FAP) are hereditary cancer predisposing syndromes characterized by increased risk of especially early-onset colorectal cancer. Predisposition to LS is caused by germline mutations in DNA mismatch repair genes leading to elevated cancer progression and microsatellite instability. FAP is associated with germline mutations in *APC* promoting cancer initiation and chromosomal instability. DNA methylation is an important epigenetic mechanism in early tumorigenesis via, e.g., field defects in non-neoplastic colon. Our aim was to study genome-wide methylation changes in colorectal specimens (adenomas and carcinomas supplemented with paired normal colon) obtained during colonoscopy surveillance, and explore the role of such alterations in tumorigenesis, with a special focus on early changes. To our best knowledge, this study is the first one to compare altered DNA methylation genome-wide in LS and FAP-associated colorectal neoplasia.

**Results:**

DNA methylation alterations were subtle in FAP adenomas, whereas in LS adenomas, changes were abundant when compared to their normal counterparts. When FAP normal and LS normal colon were compared, DNA methylation changes of FAP normal colon mirrored those occurring in LS tumors, suggesting that colorectal tumorigenesis-associated DNA methylation alterations take place already in FAP normal colon mucosa. DNA methylation age was more variable in LS than FAP normal colon, and in proximal than distal colon, when compared to individuals’ age at the time of sampling. In LS tumors, DNA methylation changes (hyper- and hypomethylation) were abundant even in adenomas with low-grade dysplasia and stable microsatellites and peaked in adenomas with high-grade dysplasia. LINE-1 hypomethylation was more prominent in LS adenomas than FAP adenomas, but normal colon of LS and FAP displayed similar levels of LINE-1 methylation.

**Conclusions:**

Genome-wide DNA methylation changes are an integral part of FAP and LS-associated colorectal tumorigenesis. Occurrence at early stages, even in non-neoplastic colonic mucosa, and increased prevalence with progressive dysplasia suggest a role in tumor development. Overlap of many of the topmost DNA methylation alterations between LS and FAP, and previous reports of their occurrence in sporadic colorectal and other tumors as well, imply their broad biological relevance and possible biomarker potential for clinical applications.

**Supplementary Information:**

The online version contains supplementary material available at 10.1186/s13148-025-01940-x.

## Background

Familial colorectal cancer comprises approximately 25–35% of all colorectal cancer (CRC) cases, and approximately 10% of CRCs have a hereditary basis [1, 2]. Lynch syndrome (LS) and familial adenomatous polyposis (FAP) are among the most common hereditary cancer predisposing syndromes. LS and FAP are autosomal dominant conditions characterized by increased risk of CRC as well as other tumors such as endometrial, ovarian, and (extracolonic) gastrointestinal tumors in LS [3], and duodenal and fundic gland polyps in FAP [4, 5]. Predisposition to LS is due to germline mutations in DNA mismatch repair (MMR) genes (*MLH1*, *MSH2*, *MSH6*, and *PMS2*) [6] or deletion in *EPCAM* gene leading to transcriptional silencing by *MSH2* hypermethylation [7], whereas germline mutations in the *APC* gene underlie FAP [8]. Three pathways exist in colorectal carcinogenesis: microsatellite instability (MSI), chromosomal instability (CIN), and CpG Island Methylator Phenotype (CIMP) [9]. LS represents the MSI pathway and elevated cancer progression, and FAP is characterized by CIN and elevated cancer initiation.

Colorectal adenomas are noninfiltrative tumors with different grades of dysplasia that may develop into CRC. In LS, the cumulative number of adenomas generally remains below ten [10]. It has become evident that the predisposing gene affects the risk of development of advanced adenomas and cancer incidence, the first being the highest for *MSH2* carriers [11], and the latter for *MLH1* and *MSH2* carriers [3, 12]. Traditionally, CRC is thought to emerge through the adenoma-carcinoma pathway, but growing evidence shows that it is not applicable to all LS cases: regular and frequent colonoscopy surveillance and removal of colorectal adenomas have not significantly decreased the lifetime risk of CRC in *MLH1* and *MSH2* carriers [3].

Accumulation of point mutations, especially insertion/deletion mutations (indels) at repetitive sequences due to defective MMR, gives rise to MSI as a characteristic feature of LS tumors. The MMR system is impaired after a loss of the second functional allele of the predisposing gene. Approximately, 75% of LS-associated colorectal adenomas are MMR-deficient [13]. Also, morphologically normal colon mucosa of LS carriers can harbor MMR-deficient crypts displaying MSI and mutations in tumor suppressor genes, suggesting the crypts can develop into cancer [14, 15]. This may explain the occurrence of CRC despite frequent surveillance. Also, tumors may grow in polypoid, flat, or depressed manner [16], of which two latter ones may be difficult to detect at early stages during surveillance. Taken together, the current knowledge suggests that LS CRC can develop either from MMR-deficient crypts, or from MMR-proficient or MMR-deficient adenomas [11, 13].

Multiple usually concurrent colorectal adenomas and polyps characterize FAP [8]. Polyposis is defined as more than ten cumulative adenomas, which is unusual in LS, whereas classical FAP typically manifests as ≥ 100 adenomas in the colorectum. FAP phenotype depends on the genomic position of the germline *APC* mutation [17, 18]. Certain loci are associated with attenuated FAP (aFAP) characterized by less than 100 cumulative adenomas, and a lifetime risk of CRC around 70% [19], whereas for classical FAP the CRC risk is 100% if left untreated [20]. Thus, prophylactic colectomy is a standard cancer-preventative method normally at early adulthood [18]. *APC* is a tumor suppressor gene (“gatekeeper”), and truncating mutations lead to accumulation of β-catenin in the cytoplasm, disrupting WNT signaling, cell homeostasis, and chromosome segregation and promoting aneuploidy and CIN [8, 21].

From a technical standpoint, determining which cancer precursor might evolve into cancer is highly challenging, if not impossible. Nonetheless, delving into these potential cancer precursors can illuminate the initial stages of tumorigenesis. The role and timing of cancer-associated mutations, such as those in *APC* and *CTNNB1*, in LS-associated tumorigenesis have been studied recently [11]. Besides mutations, epigenetic changes and disruption of epigenetic mechanisms have been identified as important regulators of cancer initiation and progression [22]. Moreover, versatile mechanisms of interplay between genome and epigenome in CRC have unfolded recently [23]. DNAm age is a recognized phenomenon across different tissues and a biomarker of biological aging and disease [24–26]. It may be influenced by genetic cancer predisposition; for example, blood from a subset of LS individuals [27] and colon organoids derived from FAP patients [28] may display accelerated DNAm age compared to healthy subjects. We observed DNA methylation changes appearing already in LS adenomas with low-grade dysplasia with retained MMR expression and stable microsatellites [29]. Our previous studies have shown that CIMP is a relatively common event in LS tumors increasing along dysplasia [29–31]. This drew our attention to DNA methylation as an early tumorigenic factor. The aim of the current investigation was to study genome-wide DNA methylation changes in colorectal adenomas and CRCs derived from two different cancer-predisposition conditions, LS and FAP, and to assess how DNA methylation alterations are related to the different characteristics of those two hereditary tumor groups. To our best knowledge, this study represents the first exploration of genome-wide DNA methylation changes within the context of comparing LS and FAP-associated colorectal tumors.

## Methods

### Study design

**Fig. 1:**
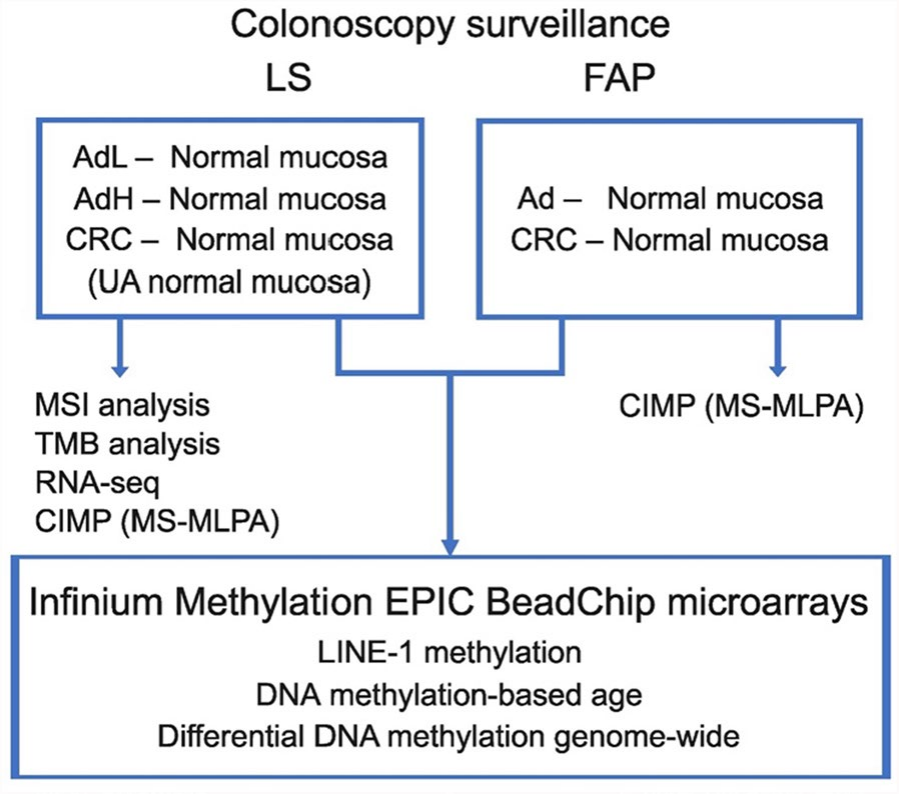
Study design. Colorectal adenomas (Ad) and carcinomas (CRC) were collected from colonoscopy surveillance of LS and FAP carriers and included in molecular analyses shown (see Methods below for details)

We used an unselected fresh frozen sample set collected prospectively from LS and FAP carriers during their regular colonoscopy surveillance (Fig. 1). We selected all tumors (adenomas and carcinomas) from LS patients paired to normal colon tissue, and all adenomas collected from FAP patients during the first sampling round paired to normal colon tissue. LS adenomas were divided into those with low-grade dysplasia (AdL) and those with high-grade dysplasia (AdH). Besides, from LS carriers, we selected a normal sample set collected from individuals with no previous or present tumor diagnoses (UA normal mucosa). Our aim was to study genome-wide methylation profiles of these tumors compared to paired normal tissue and characterize plausible recurrent alterations, especially those present already in cancer precursors and further also in carcinomas.

### Samples

Colorectal samples originated from LS (*n* = 34) and FAP (*n* = 30) patients with verified germline mutation in MMR genes (*MLH1*, *MSH2* or *MSH6*) or *APC*, respectively. Sampling was done prospectively in three different time points between 2012 and 2022 at the Helsinki University Hospital, Helsinki, Finland, during the patients’ regular colonoscopy surveillance. Biopsies were fresh frozen in liquid nitrogen. Normal colon mucosa biopsies were collected and supplemented with tumorous material whenever possible after diagnostic purposes. Normal mucosa samples were constantly taken from caecum, transverse colon, and sigmoid colon, and from the same colon sublocation as the possible lesion. Proximal colon was defined from caecum to transverse colon, and distal colon from descending colon to rectum.

Of the LS sample set, tumor samples were confirmed by a pathologist as adenomas with low or high-grade dysplasia, or carcinomas. Tumor samples were paired with normal mucosa samples from the same time point and the same or closest colon sublocation, avoiding the immediate proximity of the tumor. The normal mucosa sample set paired to tumors is referred to as LS normal samples (LS normal). We also included a normal colon sample set collected from LS individuals who had no history of any colorectal polyps, adenomas, or carcinomas. These are referred to as normal samples of unaffected LS individuals, i.e., LS unaffected normal (LS UA normal). Sample characteristics are presented in Table 1, and Additional Table 1.

**Table 1:** Characteristics of the samples

	LS UA normal	LS normal	LS AdL	LS AdH	LS carcinoma	FAP normal	FAP polyp/adenoma	FAP carcinoma
No. of patients	9	23	15	7	5	30	25	1
No. of samples	16	32	21	7	5	31	25	1
Male sex	8 (50%)	22 (69%)	15 (71%)	4 (57%)	3 (60%)	12 (39%)	11 (44%)	0
Age at diagnosis, years (mean ± SD (range))	39.4 ± 7.2 (32–52)	54.6 ± 10.7 (33–75)	54.4 ± 10.3 (33–73)	55.1 ± 12.0 (33–75)	56.2 ± 9.7 (46–74)	49.8 ± 16.6 (18–80)	48.5 ± 16.3 (18–80)	70
*Germline mutation in*								
*MLH1*	12 (75%)	24 (75%)	16 (76%)	5 (71%)	4 (80%)	NA	NA	NA
*MSH2*	4 (25%)	4 (12.5%)	2 (10%)	1 (14%)	1 (20%)	NA	NA	NA
*MSH6*	0	4 (12.5%)	3 (14%)	1 (14%)	0	NA	NA	NA
*Sample location*								
Proximal	9 (56%)	25 (78%)	14 (67%)	4 (57%)	4 (80%)	0	0	0
Distal	7 (44%)	7 (22%)^1^	7 (33%)	3 (43%)	1 (20%)	31 (100%)	25 (100%)	1
MSI	NA	NA	11 (54%)	5 (71%)	5 (100%)	NA	NA	NA
CIMP(+)	NA	NA	4 (19%)	1 (14%)	2 (40%)	NA	0	1

^1^Four distal (descending colon) tumors were paired with proximal (transverse colon) normal mucosa sample, as the distal sample was collected at very close proximity of the tumor

Proportions are calculated based on number of the samples. Three LS patients had adenomas with low-grade dysplasia in two different time points. One LS patient had both adenoma with low- and high-grade dysplasia

AdH, adenoma with high-grade dysplasia; AdL, adenoma with low-grade dysplasia; CIMP, CpG island methylator phenotype; FAP, familial adenomatous polyposis; LS, Lynch syndrome

Of the FAP sample set, we selected all the normal samples collected during the first round (plus one normal sample paired to carcinoma from the sampling round two) and paired polyp/adenoma samples whenever there was an indication of polyp or adenoma in the colonoscopy or pathology report (the latter one was not available for each polyp sample) (Table 1; Additional Table 1). None of the FAP patients had any indication of a continuous layer of colonic polyps.

Of 140 samples, two samples failed in the methylation array analysis: one LS normal sample paired to adenoma with low-grade dysplasia, and one FAP carcinoma sample. Thus, there is one LS normal sample less than LS tumorous samples.

### DNA and RNA extraction

Total DNA and RNA were isolated with the Qiagen AllPrep DNA/RNA Mini Kit (Qiagen, Valencia, CA). Nucleic acids were quantified with the Qubit fluorometer (Thermo Fisher Scientific, Waltham, MA). RNA quality was assayed with the Agilent 2100 Bioanalyzer (Agilent Technologies, Santa Clara, CA).

### MSI analysis

Tumor DNA samples were assayed with two mononucleotide repeat markers, *BAT25* and *BAT26*. Both markers have been shown to be sensitive and specific markers of high-degree MSI [27–29]. Tumors were considered MSI if at least one of the markers indicated MSI, and MSS if both markers were stable.

### CIMP analysis

Samples underwent CIMP analysis with methylation-specific multiplex ligation-dependent probe amplification using the SALSA MLPA probemix ME042-B2/C1/C2 (MRC Holland, Amsterdam, The Netherlands), as described previously [29]. According to the Weisenberger panel, a sample was considered CIMP positive if three out of five genes were hypermethylated [32].

### DNA methylation array and preprocessing

Genome-wide DNA methylation was studied using the Infinium MethylationEPIC BeadChip microarray (Illumina, San Diego, CA) following the manufacturer’s standard protocol. DNA of 500–1000 ng was used for bisulfite conversion with the Zymo EZ DNA methylation Kit (Zymo Research, Irvine, CA) followed by DNA methylation quantification.

All the data processing and analyses were performed using the R software (v.4.2.2). First, IDAT files were processed with minfi package (v.1.44.0) [33]. IlluminaHumanMethylationEPICanno.ilm10b5.hg38 was used as a manifest file for processing the EPIC data. Gene annotations were done using the UCSC_RefGene_Name in the EPIC manifest. All genomic coordinates are based on the reference genome of hg19. Quality control was performed with minfi and shinyMethyl (v.1.34.0) packages [34]. Raw data was normalized using the functional normalization (preprocessFunnorm in minfi) since it is applicable in normal-cancer comparisons where global changes can be expected [35]. This data was used for comparisons between tumorous samples and paired normal samples. Normal samples also underwent quantile normalization (preprocessQuantile in minfi) which is applicable on samples when global changes are not expected [33, 36], and the produced data was used for the comparisons between normal samples.

Probes were filtered out with following criteria: detection *P* value less than 0.01 in one or more samples, cross reactive probes as suggested by Pidsley et al. and McCartney et al. (function xreactive_probes in maxprobes, v.0.0.2) [37, 38], probes located in X and Y chromosomes, non-CpG probes (function dropMethylationLoci in minfi), SNP-containing probes as suggested by Zhou et al. using options MASK_general = TRUE and MASK_general_FIN = TRUE, as all our sample material was collected from Finnish patients [39]. Produced *M* and beta (*β*) values were used in subsequential analysis steps, as specified.

### DNA methylation age prediction

DNA methylation (DNAm) age of the samples was predicted using Horvath’s coefficients (agep in wateRmelon, v.2.4.0) [24, 26, 40]. DNAm age was compared to individuals’ age at the time of the tissue sampling using the Spearman’s correlation test and calculating the difference value (DNAm age minus age at the sampling) for each normal colon sample and investigating those values across the normal sample groups.

### Differential methylation analysis

*M* values were used for the statistical analysis of differentially methylated probes (DMPs) assayed with limma package (v.3.54.2) [41], including individual’s age and colon sublocation (in LS samples, division into proximal and distal, and in FAP samples division into two groups: one including rectal specimens and another one specimens from descending and sigmoid colon) as covariables, because both age [25], and colonic location [42] are factors affecting DNA methylation. *β* values were produced with getBeta function in minfi package, and average *β* values were calculated for each sample group. The probes with Benjamini-Hochberg (BH)-adjusted* P* value < 0.01 calculated from *M* values and |Δ*β*|> 0.1 (LS normal vs. LS adenoma with low grade dysplasia, FAP normal vs. FAP polyp, and all comparisons between normal samples) or 0.15 (LS normal vs. LS adenoma with high-grade dysplasia and LS carcinoma) were considered DMPs.

The DMPs ranked by the largest log2 fold change of *M* values were used as input for analysis of differentially methylated promoters using mCSEA package (v.1.18.0) with default settings for promoters (minimum number of CpGs = 5, if not otherwise stated) (mCSEATest function) [43]. Promoter region was considered significant if BH-adjusted *P* value (i.e., false discovery rate (FDR) value) was less than 0.05. The DMPs fulfilling aforementioned cut-off values were used as an input to analyze Gene Ontologies (GO), KEGG pathways, and gene sets (GSA) using the missMethyl package (v.1.32.1, functions gometh and gsameth; genomic.features set as 5′UTR/TSS1500/TSS200/1stExon, in gsameth collection was based on hallmarks in the Molecular Signatures Database v.7.1 [44]. GO biological process (BP) ontologies with FDR or *P* value less than 0.05 (specified in the Results, which one was used) were reduced by using the Revigo tool (package rrvvgo, v.1.14.1) with default threshold of 0.7 and org.Hs.eg.db as a database [45].

Differentially methylated regions (DMRs) were studied with DMRcate (v.2.12.0) [46] default settings (lambda = 1000, C = 2) using the limma design of the DMP analysis. Cut-off values for DMRs were: BH-corrected Stouffer’s *P* value < 0.05 and mean |Δ*β*|> 0.10 or 0.15, as specified above for DMPs, unless otherwise specified in the Results.

### Hypermethylation analysis for tumors

CpG island (CGI)-associated promoter (hyper)methylation level for each tumor sample was determined by comparing them to paired normal samples by selecting the probes from the DMP analysis (see details above), as follows. For LS tumors, the probes that were commonly hypermethylated in all LS tumor groups, located in CGIs and at least in one of gene regions of 5′UTR/TSS200/TSS1500/1stExon (*n* = 49) were selected. Similarly, for FAP adenomas, the probes that were hypermethylated in FAP adenomas compared to normal counterparts, located in CGIs and gene regions of 5′UTR/TSS200/TSS1500/1stExon (*n* = 21) were selected. Average *β* value of selected probes was calculated for each tumor sample separately. In LS tumors, higher average *β* values calculated by this method were associated with positive CIMP status (0.32 ± 0.14 vs. 0.48 ± 0.12, Wilcoxon test *P* value = 0.012), confirming its accuracy when evaluating CGI hypermethylation in the samples.

### LINE-1 hypomethylation analysis

To study long interspersed element 1 (LINE-1) hypomethylation as a surrogate marker for global hypomethylation, we retrieved the genomic locations of evolutionary young LINE-1 (L1HS and L1PA with plausible retrotransposition capability) subfamilies from the RepeatMasker of UCSC Genome Browser database (hg19) and defined the EPIC probes mapping to the selected LINE-1 elements, following the method presented in recent studies [47, 48]. *β* values for those probes were averaged for each sample and were used as LINE-1 methylation value in the downstream analyses.

### Parallel targeted sequencing

In an ongoing study, we have performed parallel targeted sequencing on tumorous and blood samples from patients with LS using the Pan Cancer panel with nearly 1000 cancer-associated genes and intronic hot spots (unpublished data). Sequencing was conducted and data analyzed as described previously [49]. The tumor samples’ mutation burden (TMB) was determined and used for the present purposes.

Somatic mutations were called with VarScan2 v2.3.2 [50] with default parameters, and Fisher's exact test *P* value of tumor versus normal for somatic/loss of heterozygosity calls less than 0.01 was considered significant. Variant impact prediction was done with SnpEff v4.0 and Ensembl v68. Variant was accepted for analysis if it was a nonsynonymous mutation, with variant allele frequency more than 5% and got PASS status from Dragen (analysis pipeline v3.9; Illumina, San Diego, CA). Tumor mutation burden was calculated as the number of significant somatic mutations/6.4 mega base (Mb). A tumor was considered hypermutated if it harbored more than ten significant somatic mutations per Mb.

### RNA sequencing

In total, 36 RNA samples from LS patients underwent RNA sequencing in our previous study [51] and overlapped with the sample material used in the present methylation array assay. The samples consisted of normal samples from unaffected LS carriers (*n* = 14), normal samples (*n* = 10), adenomas with low-grade dysplasia (*n* = 4), adenomas with high-grade dysplasia (*n* = 4), and carcinomas (*n* = 4) (Additional Table 1).

RNA sequencing and analysis were done, as described previously [51]. Briefly, the sequencing libraries were done using the Illumina TruSeq stranded mRNA-kit with RNA input of 500 ng, and they were sequenced with the Illumina Novaseq 6000 system in two runs. RNA-seq data was preprocessed followingly: adapter sequences were removed using Trimmomatic [52], reads were aligned using STAR [53], and genome annotations from Ensembl v68 were used to convert alignments to counts with HTSEQ (‘Intersection Strict’ setting for overlapping sequence features) [54]. The ComBat method was used for the correction for two different sequencing runs [55]. The differential expression analysis was done using the DESeq2 [56]. Differential expression was considered significant if shrinkage T test-based BH-adjusted *P* value was less than 0.05 [57].* P* values were estimated by fitting a normal distribution to the permuted results.

### Comparison of DNA methylation and gene expression

The RNA-seq expression values of 36 samples were correlated for the selected groups of probes and genes. First, the averaged *β* values were calculated selecting probes, as follows: resulted leading-edge probes (i.e., those that contributed most for differentially methylated promoters in mCSEATest promoter analysis) in LS tumor groups compared to normal counterparts, and for common differentially methylated promoters in all LS tumors groups, the common leading-edge probes in all LS tumor groups were selected. Second, DMRs overlapping with genes were tested similarly, we selected probes that were within DMRs and averaged the *β* values. Those were compared with expression values using the Spearman’s correlation test. The negative correlation (*R* value) with BH-adjusted *P* value < 0.05 was considered significant.

## Results

### Mutation burden and MSI in LS tumors

For LS tumors, targeted parallel sequencing data were available to investigate the tumor mutation burden (TMB). When dividing tumors into histological groups, hypermutated (TMB > 10 mutations by Mb) tumors were observed followingly: 10/21 (48%) adenomas with low-grade dysplasia (TMB range 0–25.6), 5/7 (71%) adenomas with high-grade dysplasia (TMB range 0–26.4), and 4/5 (80%) carcinomas (TMB range 7.3–39.1) were hypermutated (Fisher’s exact test *P* value = 0.34). Of 33 tumors, 19 (58%) were MSI and hypermutated (TMB range 11.9–39.1), two (6%) were MSI but not hypermutated (TMBs 7.3 and 8.6), and all MSS tumors (36%) were non-hypermutated (TMB range 0–3.1) indicating an evident connection between MSI and increased TMB (Fisher’s exact test *P* value = 2.6e−07). TMB values are presented in Additional Table 1.

### LINE-1 hypomethylation, hypermethylated DMPs, and TMB

**Fig. 2:**
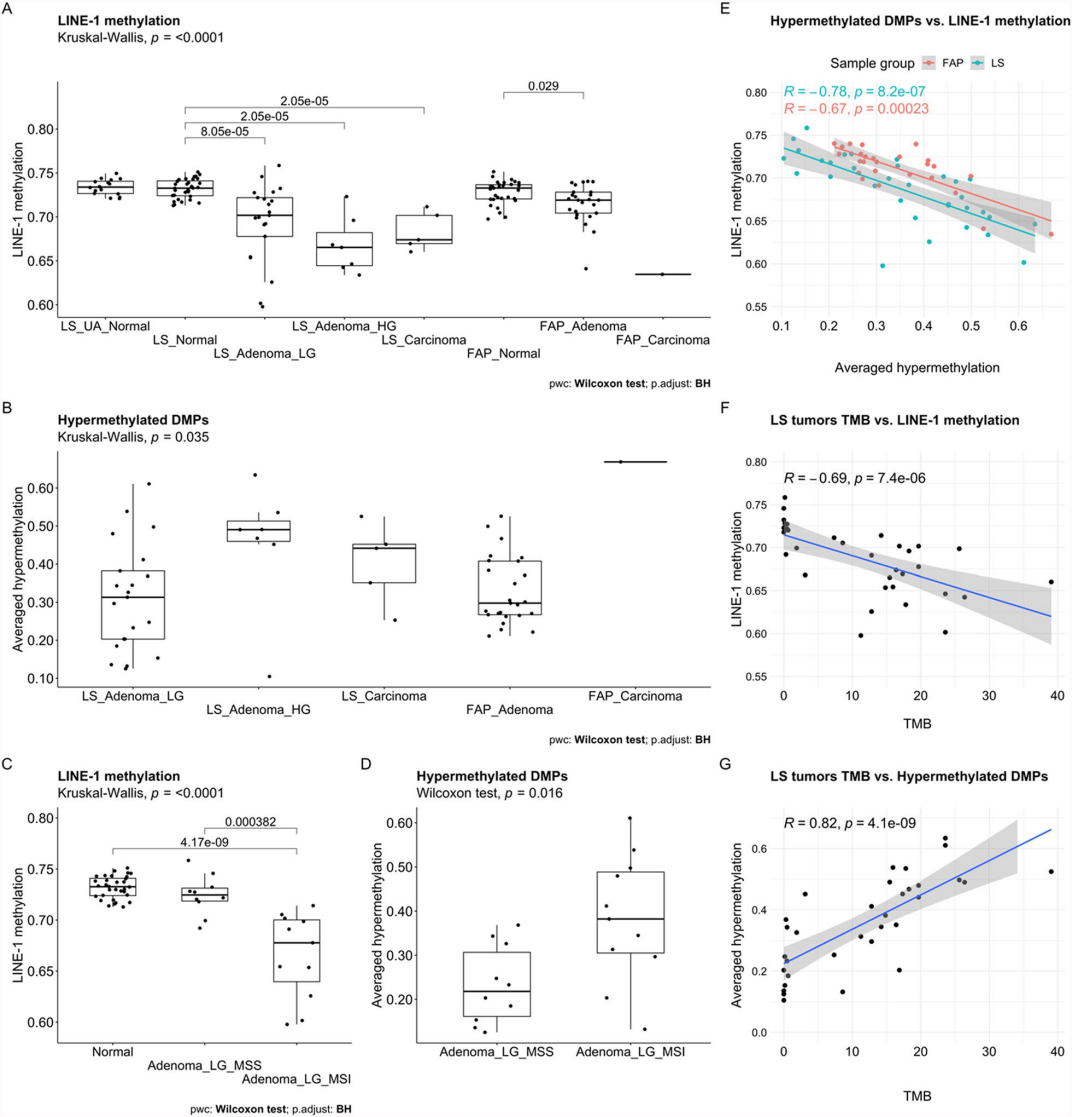
LINE-1 methylation across the samples, averaged hypermethylation of tumorous samples, and TMB of LS tumors. **A** LINE-1 methylation across all the samples. Kruskal–Wallis test was applied on averaged LINE-1 methylation *β* values of each sample group (*P* = 7.7e−09). Pairwise comparisons were studied using the Wilcoxon test and BH-adjusted *P* values < 0.05 are presented in the figure. **B** Averaged *β* values of hypermethylated probes (see Materials and Methods, and Results) in LS and FAP tumor samples. Kruskal–Wallis test was applied on all the sample groups, and Wilcoxon test for pairwise comparisons of LS tumor sample groups were applied, but all BH-adjusted *P* values were > 0.05. **C** LINE-1 methylation levels in LS normal and MSS and MSI adenomas with low-grade dysplasia. Kruskal–Wallis test was applied on all the samples (*P* = 3.69e−06). Pairwise comparisons were studied using the Wilcoxon test and BH-adjusted *P* values < 0.05 are presented in the figure. **D** Averaged *β* values of hypermethylated probes (see Materials and Methods, and Results) in MSS and MSI LS adenomas with low-grade dysplasia. In the boxplots, the upper and lower edges of the boxes indicate the 75th and 25th percentiles, the horizontal line inside the box denotes the median, and the whiskers indicate the lowest and highest values (outliers are shown outside the whiskers). Each sample’s averaged *β* value is indicated as a single dot.** E** LINE-1 methylation level compared to averaged hypermethylation in LS and FAP tumors using the Spearman’s correlation test. **F** TMB compared to LINE-1 methylation level in LS tumors using the Spearman-correlation test. **G**. TMB compared to averaged hypermethylation in LS tumors using the Spearman’s correlation test. The shaded areas represent 95% confidence intervals

LINE-1 hypomethylation status was studied in LS and FAP by averaging methylation values of selected young LINE-1-associated probes. LINE-1 methylation level is a surrogate marker for global hypomethylation, and we investigated the methylation levels across the samples (Fig. 2A). LS normal samples were compared to LS unaffected normal samples, LS tumors, and FAP normal, LS adenomas with low-grade dysplasia were compared to FAP adenomas, and FAP normal samples were compared to FAP adenomas (ns Wilcoxon test BH-adjusted *P* values). Normal samples across all groups had similar levels of methylation. LINE-1 hypomethylation was evident in all tumors compared to their normal counterparts. Among LS tumors, adenomas with high-grade dysplasia revealed the highest degrees of LINE-1 hypomethylation, although the differences in average values were small (0.69 ± 0.04, 0.67 ± 0.03, and 0.68 ± 0.02, in adenomas with low-grade dysplasia, adenomas with high-grade dysplasia, and carcinomas, respectively). The average degree of LINE-1 methylation in LS normal mucosae was 0.73 ± 0.01 (significantly higher than in the paired tumor groups). FAP adenomas showed more subtle differences relative to paired normal samples (0.71 ± 0.02 vs. 0.73 ± 0.01, respectively, BH-adjusted Wilcoxon test* P* = 0.029). Three LS adenomas with low-grade dysplasia (all MSI, and one CIMP positive) displayed very low LINE-1 methylation values (< 0.65), complying with our previous study, where we observed methylation alterations already in MSS LS adenomas with low-grade dysplasia [29].

CpG island and promoter-associated hypermethylation for each tumor sample was calculated as presented in Methods. Figure 2B illustrates these averaged hypermethylation values in all tumor sample groups. LS adenomas with low-grade dysplasia generally showed lower values compared to adenomas with high-grade dysplasia (0.31 ± 0.14 vs. 0.45 ± 0.16, respectively) and carcinomas (0.40 ± 0.10), but the pairwise comparisons did not reach statistical significance by BH-adjusted Wilcoxon test. Hypermethylation levels of FAP adenomas (0.33 ± 0.09) were comparable to LS tumors (with a suggestive difference when compared to LS adenomas with high-grade dysplasia: Wilcoxon test BH-adjusted *P* = 0.092). A single FAP carcinoma showed a very high averaged hypermethylation value (0.67) and was higher than in FAP adenomas and LS carcinomas, but the difference was not statistically significant.

In the present study, LS adenomas with low-grade dysplasia were divided into MSS (*n* = 10) and MSI (*n* = 11) subgroups. MSS adenomas showed somewhat lower LINE-1 methylation vs. normal mucosa (0.72 ± 0.02 vs. 0.73 ± 0.01, BH-adjusted Wilcoxon test *P* value = ns), but the difference was more pronounced in MSI adenomas (0.67 ± 0.04 vs. 0.73 ± 0.01, BH-adjusted Wilcoxon test *P* = 4.17e−09) (Fig. 2C). Furthermore, MSS adenomas displayed milder hypermethylation than MSI adenomas (0.23 ± 0.09 vs. 0.38 ± 0.14, Wilcoxon test BH-adjusted *P* = 0.016; Fig. 2D), although a few MSS adenomas showed somewhat higher degrees of hypermethylation (above 0.30 in three samples).

Consistently, statistically significant negative correlation was evident between averaged hypermethylation and LINE-1 methylation levels both in LS (*R* = − 0.78, Spearman’s test *P* = 8.2e−07; Fig. 2E) and FAP (*R* = − 0.67, Spearman’s test *P* = 0.0002) tumors. TMB data was available for LS tumors, and there was clear inverse correlation between TMB and LINE-1 methylation level (*R* = − 0.69, Spearman’s test *P* = 7.4e−06; Fig. 1F), and evident positive correlation between TMB and averaged hypermethylation (*R* = 0.82, Spearman’s test *P* = 4.1e−09; Fig. 2G). Taken together, increased TMB in LS tumors correlates positively with hypermethylation of DMPs and LINE-1 hypomethylation, and in LS and FAP tumors, LINE-1 methylation level decreases along higher CGI and promoter-associated hypermethylation.

### DNA methylation-based predicted age

**Fig. 3:**
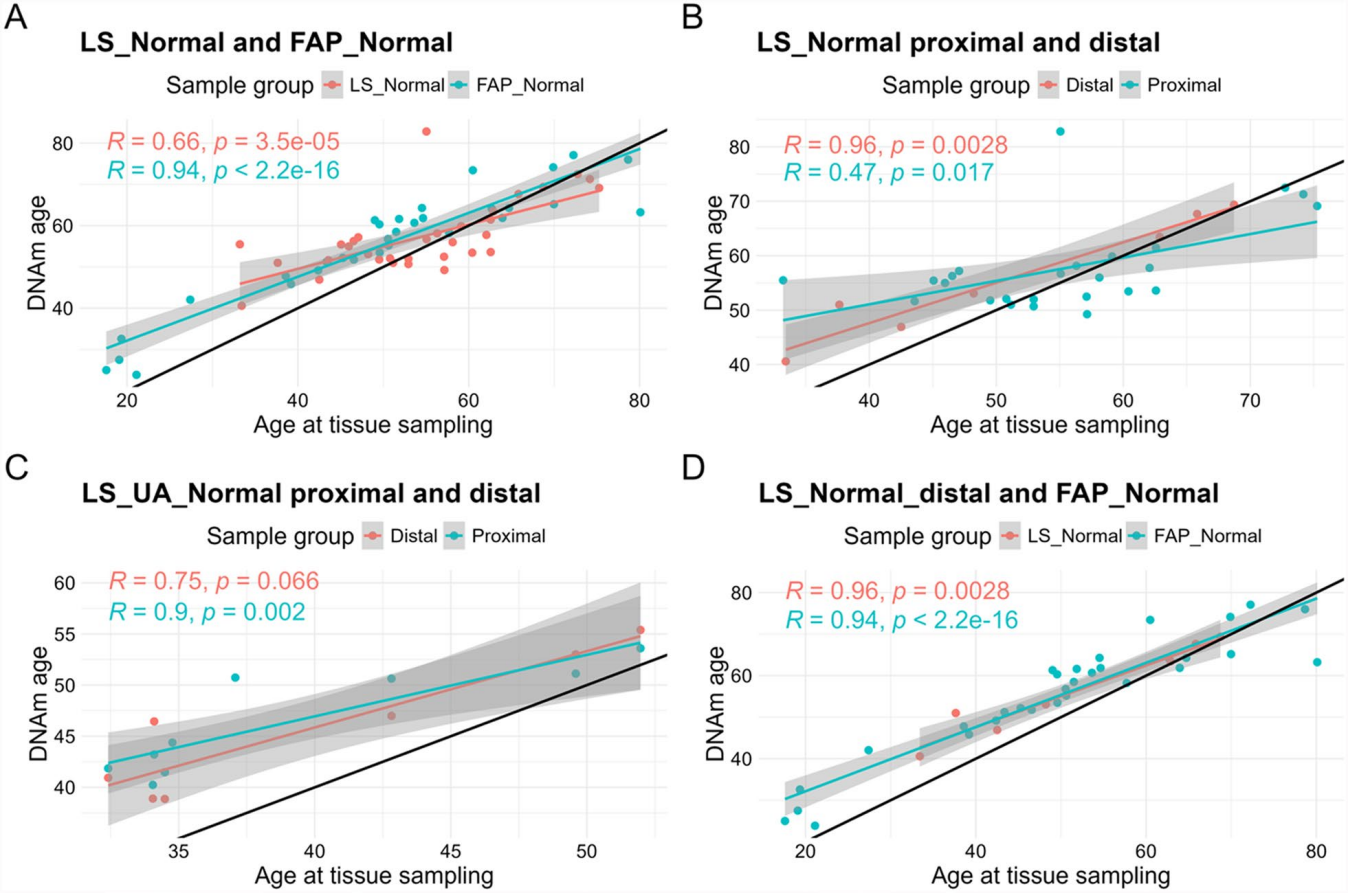
DNAm age and age at the sampling. **A** DNAm age compared to age at the sampling in LS normal, LS unaffected normal, and FAP normal samples. Black line denotes the bisector line when y = x. **B** DNAm age compared to age at the time of tissue sampling in LS normal samples divided into proximal and distal samples. **C** DNAm age compared to age at the sampling in LS unaffected normal samples divided into proximal and distal samples. **D** DNAm age compared to age at the sampling in distal samples of LS normal and FAP normal. Each sample’s averaged *β* value is indicated as a single dot

Horvath’s epigenetic clock was used to estimate DNAm age of colon normal samples [24, 26]. First, correlation tests between DNAm age and age at the sampling of normal mucosae suggested that DNAm age of FAP patients’ normal colon was most concordant with patients’ age at the sampling (Spearman’s test *R* = 0.94, *P* < 2.2e−16; Fig. 3A). The normal mucosa of LS patients younger than 50 years old seemed to have accelerated DNAm age compared to older LS patients, who displayed lower DNAm age compared to their age at the sampling (average difference values 9.2 ± 5.3 vs. − 0.59 ± 7.3, respectively, Wilcoxon test *P* value = 3.06e−06; Spearman’s test *R* = 0.66, *P* = 3.5e−05; Fig. 3A). Normal colon mucosa of unaffected LS cases had somewhat more straightforward correlation (Spearman’s test *R* = 0.85, *P* = 2.9e−05; Fig. 3A), but all unaffected individuals were less than 55 years old, so unfortunately, we cannot draw conclusions on methylation changes in older individuals.

As different colon sublocations (78% of LS normal colon samples were of proximal origin and all FAP samples of distal origin) possibly contributed to the methylation difference between LS and FAP samples, we compared proximal and distal normal samples of LS patients (Fig. 3B, C). Indeed, correlations were somewhat different in proximal and distal colon, although the number of distal LS normal samples was low (*n* = 7 for both LS normal and LS UA normal). DNAm age of distal LS normal samples was more concordant with patients’ age at the sampling (Spearman’s test *R* = 0.96, *P* = 0.003), and was somewhat accelerated in proximal samples of younger LS patients (average difference values for patients under and over 50 years: 10.3 ± 6.0 vs. − 0.86 ± 8.1, respectively, Wilcoxon test *P* value = 0.0002; Spearman’s test *R* = 0.47, *P* = 0.017; Fig. 3B). In unaffected LS carriers, the proximal (Spearman’s test *R* = 0.9, *P* = 0.002) and distal (Spearman’s test *R* = 0.75, *P* = 0.066) colon showed more similar correlations (Fig. 3C). Correlations between DNAm age and age at the sampling were very similar in distal LS normal samples and FAP normal samples as presented in the same plot in Fig. 3D. Thus, DNAm aging in normal mucosa seems to occur early, but slows down along aging of LS patients, being somewhat more prone to occur in the proximal than distal colon.

As previous tumor history may affect methylation-based aging in the normal colon mucosa, we performed the correlation tests including only those LS normal samples collected from the LS patients at the time of their first tumor diagnosis (adenoma or carcinoma; *n* = 12, of which eight were proximal and four distal samples) (Additional Fig. 1A–C). The sample size was small, and correlations did not reach statistical significance, but trends resembling the analyses above (Fig. 3A–D) were observed. It is noteworthy that there is no clear indicator why two LS normal samples represent evidently older DNAm age (difference to age at the sampling > 20 years) (Fig. 3A, B). Both were proximal normal colon mucosa samples from *MLH1* mutation carriers, who were diagnosed with adenoma with high-grade dysplasia at the sampling, males, at ages of 33 and 55, and had no previous personal history of cancer.

In general, the difference in DNAm age and age at sampling was more variable in proximal colon, with an average value near zero, whereas distal colon samples displayed a tendency of DNAm age to be higher than age at the sampling (Additional Fig. 1D).

### Overall genome-wide methylation in sample sets

Overall, when the most variable CpG sites (*n* = 1000) were selected from the filtered methylation data, histology differentiated the samples well (Additional Fig. 2, Additional Fig. 3). The majority of the FAP and LS samples were represented differently, and normal samples were mostly different compared to tumorous samples. The inherent heterogeneity of fresh-frozen sample material accounts for some of the overlap observed between normal and tumor samples.

After filtering, there were 723,636 probes for differential methylation analysis in LS sample set (LS normal and tumors). When compared to normal samples, adenomas with low-grade dysplasia showed 26,848 DMPs, adenomas with high-grade dysplasia 54,504 DMPs, and carcinomas 38,851 DMPs (numbers of DMPs out of informative probes; Chi-square *P* value < 2.2e−16; Additional Table 2). The numbers of hypo- and hypermethylated probes were 24,022 (89%) and 2,826 (11%), 44,008 (81%) and 10,496 (19%), and 22,753 (59%) and 16,098 (41%), respectively (relation of hypo- and hypermethylated probes in LS tumor groups; Chi-square *P* value < 2.2e−16). In the LS sample set, we used slightly lower *β* value threshold for adenomas with low-grade dysplasia (|Δ*β*|> 0.1 instead of > 0.15 that was applied to adenomas with high-grade dysplasia and carcinomas), to see if the changes observed in advanced tumors would be visible already on adenomas with low-grade dysplasia but plausibly with a smaller difference compared to normal counterparts. If the cut-off of |Δ*β*|> 0.15 was used, the number of DMPs was only 4331 in adenomas with low-grade dysplasia compared to normal samples.

When MSS LS adenomas with low-grade dysplasia were compared to normal samples, no DMPs were detected fulfilling the cut-offs of adjusted *P* value < 0.01, and |Δ*β*|> 0.1. If only *β* value cut-off |Δ*β*|> 0.1 was considered, 585 DMPs were observed (raw *P* value < 0.01), and of those, 156 were hypomethylated and 429 hypermethylated (Additional Fig. 4 A, B). A majority of DMPs were in CGIs (*n* = 259), and almost all of those were hypermethylated (*n* = 252) (Additional Fig. 4 A). When MSI LS adenomas with low-grade dysplasia were compared to LS normal samples or MSS LS adenomas (the cut-offs for DMPs were adjusted *P* value < 0.01 and |Δ*β*|> 0.1), the numbers of DMPs were 13,550 and 12,461, respectively. Of those, 12,828 and 722, and 12,162 and 299 were hypo- and hypermethylated, respectively (Additional Fig. 4 A, B).

**Fig. 4:**
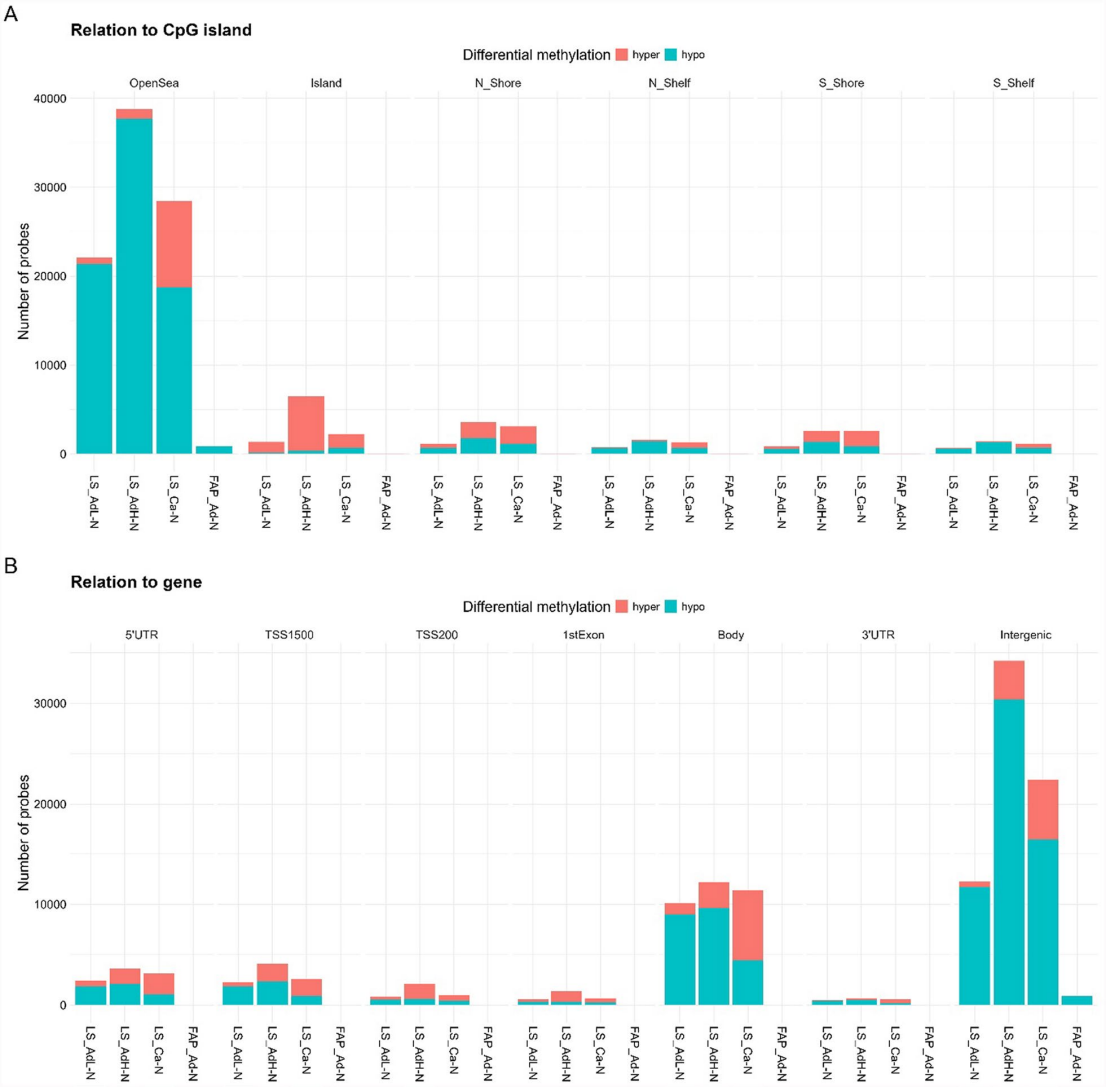
An overview of DMPs in LS and FAP tumors and their relation to CpG island and gene regions. Red color indicates hypermethylated probes and blue stands for hypomethylated probes. DMPs in **A** LS adenomas with low-grade dysplasia, LS adenomas with high-grade dysplasia, and LS carcinomas were compared to LS normal samples, and FAP adenomas were compared to FAP normal samples. **B** Overview of DMPs and their relation to gene regions

In the sample set of FAP, 725,309 probes remained after filtering for the differential methylation analysis. The comparison of normal and polyp/adenoma samples resulted in 995 DMPs, of which 917 were hypomethylated and 78 hypermethylated in adenomas (when compared to hypo- and hypermethylated probe numbers in LS adenomas with low-grade dysplasia; Chi-square *P* value = 0.008). Most of the hypermethylation in FAP adenomas occurred in CGIs (Fig. 4A).

Figure 4B presents an overview of DMPs in all tumors and their relation to gene regions. In LS tumors, hypermethylation increased with dysplasia in all gene regions. In LS carcinomas, hypermethylation events exceeded hypomethylation in all known gene regions (3′UTR, Body, 1 st Exon, TSS200, TSS1500, 5′UTR), whereas in adenomas with high-grade dysplasia, hypermethylation was only more frequent than hypomethylation in 1 st Exon and TSS200 regions, indicating increased promoter hypermethylation.

In the set of normal samples, there were 723,715 probes after filtering for all normal samples for differential methylation analysis. Methylation changes in the normal mucosa were very subtle between normal mucosae from tumor-affected and unaffected LS cases. Total number of DMPs (with thresholds of adjusted *P* value < 0.01 and mean |Δ*β*|> 0.1) was only five (Additional Table 2). When the *β* value threshold was lowered to |Δ*β*|> 0.05 (adjusted *P* value < 0.01), there were 23 DMPs. Of those, 21 (91%) DMPs were hypermethylated in LS normal compared to unaffected LS normal. In relation to CpG island, the majority (52%, *n* = 11/21) of hypermethylated probes were in Open Sea genomic region, 24% (*n* = 5/21) in S-shelves, 14% (*n* = 3/21) in N-shores, and 10% (*n* = 2) S shores. Two probes that were hypermethylated in unaffected LS normal mucosa located in CpG island. Of the DMPs, 65% (*n* = 15/23) overlapped with genic regions followingly: 47% gene body, 18% TSS1500, 18% 5′UTR, 12% TSS200, and 6% 1 st exon.

Between normal mucosa samples from LS and FAP cases, methylation changes were more prevalent: 7,284 DMPs were detected with thresholds of *P* value < 0.01 and |Δ*β*|> 0.1. Of those, 57% (*n* = 4139) were hypermethylated in LS samples compared to FAP. Like other comparisons, a majority of DMPs were in the Open Sea region (in relation to CGI) and gene body or non-gene associated gene regions (Additional Fig. 5 A, B). Here, the major difference relative to normal-tumor comparisons was more prevalent hypermethylation events in the Open Sea region. Hypermethylated DMPs occurring in CGIs were scarcer in LS samples (*n* = 144/439). In other words, differential methylation between LS normal and FAP normal consisted of most of CGI-associated DMPs being hypermethylated in FAP, and hypermethylated DMPs associated to unknown gene regions and gene bodies being more abundant in LS samples (Additional Fig. 5B). Volcano plot of the differential methylation is illustrated in Additional Fig. 5C.

Additional Fig. 6 illustrates how DMPs overlapped in different comparisons. Of DMPs in FAP adenomas compared to normal counterparts, a majority (92%, *n* = 912/995) was also present in at least one group of LS tumors (Additional Fig. 6 A). The different LS tumor groups shared 8,226 DMPs, and the proportion of shared DMPs decreased along dysplasia (Additional Fig. 6 A). Finally, DMPs between FAP normal and LS normal had much more in common with DMPs in LS adenomas than with those in FAP adenomas compared to their normal counterparts (Additional Fig. 6B).

### Differentially methylated probes in LS tumors

**Fig. 5:**
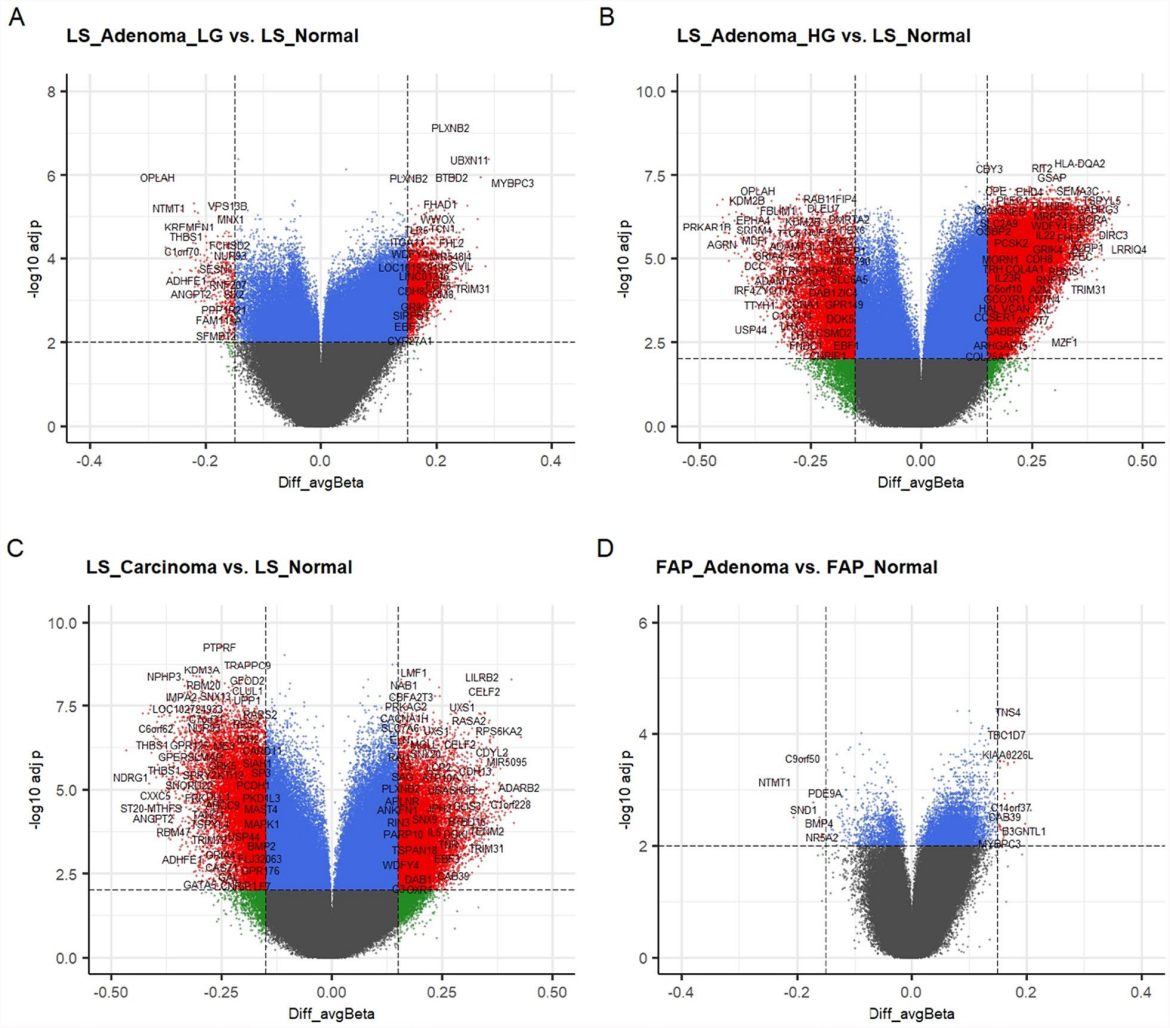
Volcano plots of DMPs in LS tumors (**A**–**C**) and FAP adenomas (**D**). Adjusted *P* values are plotted against averaged *β*-values (Diff_avgBeta). Hypermethylated probes in tumors compared to normal counterparts are represented with positive averaged *β*-values and hypomethylated probes in tumors with negative values. Red dots indicate the DMPs above both thresholds marked with dashed lines (|Δ averaged *β*|> 0.15 and BH-adjusted −Log_10_ *P* > 2, i.e. *P* value < 0.01)

Figure 5A–C shows volcano plots of DMPs in LS tumor groups. Compared to normal mucosa, methylation changes were evident already in adenomas with low-grade dysplasia, although the difference was smaller than in adenomas with high-grade dysplasia and carcinomas.

At probe-level, when selecting only those DMPs that were altered in all LS tumor groups compared to normal counterparts, there were 8226 DMPs of which 4630 were overlapping with genes (i.e., were annotated with UCSC RefSeq genes including their 5′/3′UTRs) (Additional Table 3). The genes that were represented with more than ten hypomethylated DMPs were *RORA*, *SDK1*, *CNTN4*, *PARK2*, *ADARB2*, *COL4A2*, *GPC6*, *SOX5*, *RARB*, *AMPH, GAS7*, *PTPRN2*, and *NTM*, and more than ten hypermethylated probes targeted *HOXA5*, *FBLIM1*, and *NR5A2*. When considering CGI- and 5′UTR/TSS200/TSS1500/1stExon-associated probes, 186 DMPs remained. When among those at least three probes targeting the same gene was set as a requirement, targeted genes were *ADHFE1*, *CDH3, CDO1, DOK6, EYA4, FBLIM1, GRIA4, HOXA4, ITGA4, KLHDC7B, LBX2, ZNF135, ZNF471,* and ZNF542, all being hypermethylated in tumors (Additional Table 3). If only DMPs with |Δ*β*|> 0.20 in all tumors were considered, there were 251 DMPs, of which 145 overlapped with a gene. The 10 probes with the largest average of |Δ*β*| of all tumors are presented in Table 2A.

**Table 2:** Top ten probes with the highest methylation difference (|Δ averaged *β*|) and overlapping with gene. A. Common DMPs in all LS tumor groups compared to LS normal. BH-adjusted *P* values can be found in the Additional Table 3 for each LS normal-LS tumor comparison. B. DMPs in FAP adenomas compared to FAP normal. C. DMPs in LS normal compared to FAP normal

A	Chr	Position	Probe	Gene	Relation to gene	Relation to island	Normal avgBeta	Adenoma LG avgBeta	Adenoma HG avgBeta	Carcinoma avgBeta
	chr11	47359017	cg14642259	*MYBPC3*	Body	N_Shore	0.91	0.58	0.38	0.50
	chr1	1475265	cg08738570	*C1orf70*	Body	N_Shore	0.10	0.37	0.62	0.50
	chr17	81006524	cg10344477	*B3GNTL1*	Body	N_Shelf	0.64	0.35	0.21	0.23
	chr8	145106582	cg26256223	*OPLAH*	Body	Island	0.05	0.29	0.56	0.42
	chr8	145106438	cg17301223	*OPLAH*	Body	Island	0.05	0.28	0.58	0.41
	chr7	127672564	cg09296001	*SND1*	Body	Island	0.06	0.33	0.49	0.47
	chr6	162603057	cg24693245	*PARK2*	Body	OpenSea	0.68	0.41	0.26	0.29
	chr8	145107012	cg22882523	*OPLAH*	Body	Island	0.11	0.39	0.51	0.48
	chr8	6420242	cg02548132	*ANGPT2; MCPH1*	1stExon; Body	N_Shelf	0.29	0.52	0.71	0.70
	chr1	200014673	cg18126097	*NR5A2*	Body	S_Shelf	0.18	0.43	0.63	0.52

B	Chr	Position	Probe	Gene	Relation to gene	Relation to island	Normal avgBeta	Adenoma avgBeta	BH-adjusted *P* value	
	chr9	132382463	cg18973112	*NTMT1; C9orf50*	TSS200; 5′UTR; 1stExon	Island	0.16	0.40	7.20e−04	
	chr12	89744150	cg27365701	*DUSP6*	Body	N_Shore	0.71	0.51	2.37e−03	
	chr20	1874527	cg19256731	*SIRPA*	TSS1500	N_Shore	0.46	0.67	3.19e−03	
	chr7	2609861	cg09159452	*IQCE*	Body	OpenSea	0.77	0.58	4.13e−03	
	chr17	81006524	cg10344477	*B3GNTL1*	Body	N_Shelf	0.52	0.33	5.43e−03	
	chr9	132382433	cg14015706	*C9orf50*	1stExon	Island	0.13	0.32	2.74e−04	
	chr7	127672564	cg09296001	*SND1*	Body	Island	0.11	0.30	2.27e−03	
	chr4	23335397	cg27343278	*MIR548AJ2*	Body	OpenSea	0.68	0.50	3.23e−04	
	chr2	182174340	cg24024260	*LOC101927156*	Body	OpenSea	0.58	0.40	2.99e−03	
	chr8	65543236	cg14155810	*CYP7B1*	Body	OpenSea	0.51	0.33	1.15e−03	

C	Chr	Position	Probe	Gene	Relation to gene	Relation to island	LS normal avgBeta	FAP normal avgBeta	BH-Adjusted *P* value	
	chr8	8748792	cg03899721	*MFHAS1*	1stExon	Island	0.89	0.44	1.48e−06	
	chr2	232262773	cg05532325	*B3GNT7*	Body	N_Shore	0.84	0.42	2.21e−07	
	chr2	232263030	cg09235217	*B3GNT7*	Body	Island	0.84	0.45	4.57e−07	
	chr10	115480523	cg07156029	*CASP7*	Body	OpenSea	0.44	0.83	1.02e−07	
	chr10	115479194	cg15770225	*CASP7*	Body	OpenSea	0.39	0.77	2.21e−06	
	chr11	102282062	cg11880242	*TMEM123*	Body	OpenSea	0.49	0.86	2.39e−08	
	chr10	115479248	cg18481049	*CASP7*	Body	OpenSea	0.46	0.83	5.49e−07	
	chr2	242585086	cg02032892	*ATG4B*	Body	OpenSea	0.83	0.47	5.77e−08	
	chr5	134401774	cg09785999	*C5orf66*	5′UTR	OpenSea	0.84	0.49	3.59e−09	
	chr13	110433784	cg24526103	*IRS2*	Body	N_Shore	0.34	0.70	4.98e−08	

All DMPs of each normal-tumor comparison were used as input and enrichment analysis was done using the GO, KEGG and GSA analyses (numerical summary is presented in Additional Table 4). DMPs located in genomic regions of TSS200, TSS1500, 1stExon, and 5′UTR were selected to analyze only those probes/genes, of which methylation occurs on the genes’ promoter region or nearby, and thus, is likely to alter gene expression. The analyses were done separately on all selected DMPs, hypomethylated and hypermethylated DMPs in tumors. Ontologies, pathways, and hallmarks with FDR < 0.05 were considered significant unless otherwise stated. Detailed lists of the enrichment analysis results are presented in Additional Table 5A–C. In LS carcinomas, although the number of hypermethylated DMPs was 16,098 (of which more than 5,000 on selected genomic regions), only one BP-ontology fulfilled a cut-off FDR < 0.05 (actin filament depolymerization). Thus, for the Revigo analysis, raw *P* value < 0.01 was used as a cut-off to select input GO terms. Similar GO results of BP-ontologies were reduced by using the Revigo tool (Additional Table 5D).

After using the Revigo tool, when all DMPs were used as an input for GO analysis, four parent terms were present in all comparisons of LS tumor groups to normal samples: G protein-coupled receptor signaling pathway, multicellular organismal process, response to stimulus, and cell recognition (Table 3; Additional Table 5D). GO BP ontologies were related to various cellular processes, indicating that DNA methylation changes affected many different pathways. Intriguingly, various inflammation-associated pathways were evidently enriched in hypomethylated DMPs in LS carcinomas (Additional Table 5D), including immune response, defense response, and leukocyte-related pathways.

**Table 3:** Common results of KEGG pathway, gene set, and reduced GO:BP analyses in all LS tumor groups (FDR < 0.05)

	Adenoma_LG		Adenoma_HG		Carcinoma	
KEGG	DE	FDR	DE	FDR	DE	FDR
Olfactory transduction	132	2.73E−31	206	1.18E−52	114	2.61E−23
Cytokine-cytokine receptor interaction	57	3.48E−06	77	3.80E−04	49	2.99E−03
Neuroactive ligand-receptor interaction	65	3.49E−06	99	2.01E−06	64	9.28E−05
Staphylococcus aureus infection	18	4.36E−02	36	9.45E−07	26	3.64E−06

GSA	DE	FDR	DE	FDR	DE	FDR
HALLMARK_INFLAMMATORY_RESPONSE	35	7.72E−03	57	8.49E−04	44	1.56E−05
**Parent terms of reduced GO:BP**	**The largest score**		**The largest score**		**The largest score**	
G protein-coupled receptor signaling pathway (GO:0007186)	28.0		39.2		15.7	
Multicellular organismal process (GO:0032501)	16.6		25.1		9.6	
Response to stimulus (GO:0050896)	10.7		12.1		14.5	
Defense response (GO:0006952)	9.5		8.3		15.6	
Response to external stimulus (GO:0009605)	7.8		9.0		10.1	
Biological process involved in interspecies Interaction between organisms (GO:0044419)	6.4		4.7		9.3	
Immune response (GO:0006955)	6.2		6.5		21.9	
Cell recognition (GO:0008037)	3.7		1.7		1.7	
Regulation of biological process (GO:0050789)	3.1		2.9		5.4	

All results are based on hypomethylated probes in tumors compared to LS normal mucosa. DE describes the number of significant genes. The largest score indicates the largest score represented of the parent term by Revigo analysis

Of KEGG pathways with FDR < 0.05, two were common in all LS tumor groups when all DMPs of each comparison were used as input: olfactory transduction, and neuroactive ligand-receptor interaction, whereas when hypomethylated DMPs as an input, two more pathways were shared: cytokine-cytokine receptor interaction, and staphylococcus aureus infection (Table 3; Additional Table 5B). Like GO analysis, hypomethylated probes in LS carcinomas suggested hypomethylation alterations in genes associated with inflammation, including Th17 cell differentiation, viral protein interaction with cytokine and cytokine receptor, and B cell receptor signaling pathways (Additional Table 5B).

GSA analysis revealed one hallmark that was enriched in all LS tumor groups, when all and hypomethylated DMPs were as an input: inflammatory response (Table 3; Additional Table 5 C). In adenomas with high-grade dysplasia, also a hallmark of epithelial to mesenchymal transition was evident with FDR < 0.05, when all and hypermethylated probes were as input (Additional Table 5 C). Different from GO and KEGG analyses, here, five hallmarks were enriched in LS carcinomas when hypermethylated DMPs were used as an input, including, e.g., KRAS signaling up (Additional Table 5 C).

### Differentially methylated regions in LS tumors

When differential methylation was studied on a larger scale as DMRs and promoters, there were 1984 (115 hypermethylated) DMRs in adenomas with low-grade dysplasia, 4560 (432 hypermethylated) in adenomas with low-grade dysplasia, and 3030 (1104 hypermethylated) in carcinomas. In total, 1253, 2930, and 2131 were overlapping with genes, respectively (Additional Table 6). There were five common DMRs (overlapping with genes) in all tumors, when selecting top 100 DMRs of each tumor group sorted by the smallest FDR value (Additional Table 6, sheet overlappingGenes_common_top100): *OR2I1P*, *OPLAH*, *NR5A2*, *MCIDAS*, and *ARHGDIB*. When selecting the common overlapping genes (i.e., gene name was present at least once in all tumors’ DMRs), there were 564, 749, and 634 DMRs in adenomas with low-grade dysplasia, adenomas with high-grade dysplasia, and carcinomas, respectively (Additional Table 6).

We averaged the *β* values of the probes that mapped within DMRs (those overlapping with genes) and ran Spearman’s correlation test on averaged *β* values and expression values of overlapping genes on LS samples with existing RNA-seq data. The numbers of DMRs overlapping with genes resulting in negative correlation (BH-adjusted *P* value < 0.05) were 141, 231, and 284 in adenomas with low-grade dysplasia, adenomas with high-grade dysplasia, and carcinomas, respectively (Additional Table 6). Of those, 41 genes were common in all normal-tumor comparisons. The ten topmost regions with the strongest negative correlation were: *DAPP1*, *PITPNC1*, *NR5A2*, *RP11-466L17.1*, *MET*, *TCN1*, *MYT1L*, *ITGBL1*, *ARHGDIB*, and *AFF1* (R value range: − 0.79 to − 0.68) (Additional Table 6).

MCEA promoter analysis revealed 63, 423, and 191 differentially methylated promoters in adenomas with low-grade dysplasia, adenomas with high-grade dysplasia, and carcinomas, respectively (Additional Table 7). Of those, 11, 45, and 53 promoters were inversely correlated with gene expression (BH-adjusted *P* value < 0.05) in LS samples, when selecting leading-edge probes to calculate averaged *β* value for LS samples with RNA-seq data and the value was correlated with gene expression value (Additional Table 7). Of all differentially methylated promoters, 22 promoters were significantly altered in all tumors (BH-adjusted *P* value < 0.05) (Table 4). Among the top of the list sorted by the smallest adjusted *P* values were five genes: *ADHFE1*, *EYA4*, *FBLIM1*, *HOXA3*, and *HOXA5*, all hypermethylated in all tumor groups. Of the 22 common promoters, we selected common leading-edge probes, i.e., those contributing most to the differential methylation in all normal-tumor comparisons for each promoter/gene, according to the mCEAtest. Of those, 21 were eligible to be compared with expression values and *ADHFE1*, *DAPP1*, *FBLIM1*, *GRIA4*, *HOXA3*, *KLF17*, and *ZNF528* revealed statistically significant negative correlation to gene expression suggesting these genes are regulated by DNA methylation and are associated to LS and/or CRC-associated tumorigenesis already at the early stages (Table 4).

**Table 4:** Differentially methylated promoters shared by all LS tumor groups (FDR < 0.05). Promoters/genes are sorted alphabetically

Promoter/gene	Average NES	Number of DMPs common	CpG island region of DMPs common	DMRs common	*β* values vs. mRNA-expression *R* value; adjusted *P* value
***ADHFE1***	3.32	9	Island, N shore	Yes	− 0.38; 0.043
*CHN2*	2.21	4	Open sea	Yes	− 0.17; 0.432
*CNTN4*	− 2.16	21	Open sea	Yes	0.56; 0.001
***DAPP1***	− 1.97	5	Open sea	Yes	− 0.79; 6.72e−06
*EYA4*	4.31	6	Island, Open sea	Only AdL, AdH	0.10; 0.683
***FBLIM1***	3.70	11	Island, N shore	Only AdH, Ca	− 0.57; 0.001
*FLI1*	1.18	5	Island, S shore, Open sea	Only Ca	NA
***GRIA4***	3.10	6	Island	Only AdH	− 0.42; 0.029
*HAPLN1*	− 2.01	7	Open sea	Yes*	0.38; 0.043
***HOXA3***	3.98	7	N shelf, N shore, S shelf, S shore	Yes	− 0.38; 0.043
*HOXA5*	4.05	14	Island, N shore	Only Ca	− 0.27; 0.169
***KLF17***	− 2.02	5	Open sea	Only AdH	− 0.66; 9.31e−05
*LBX2*	2.71	7	Island	Yes	0.03; 0.888
*LCAT*	2.81	8	Open sea	No	0.40; 0.035
*PLCH1*	2.67	6	Open sea	Only Ca	0.05; 0.843
*PREX2*	2.61	7	Island, N shore, S shelf, Open sea	Only Ca	− 0.22; 0.290
*RARB*	− 2.22	13	Open sea	Yes	0.42; 0.029
*RIMS1*	2.04	8	Island, S shore, N shore, Open sea	Only AdL, AdH	0.30; 0.131
*SEPT9*	2.14	2	Island	No	0.15; 0.493
*TBX15*	3.12	2	N shore	Only AdH	0.72; 6.85e−06
*ZFHX3*	2.34	4	N shore	Only AdH, Ca	0.00; 0.978
***ZNF528***	2.89	6	Island, N shore	Only AdH	− 0.58; 0.001

Exact statistics and details are presented in Additional Table 7. Average NES indicates the average value of LS tumors’ normalized enrichment score by the number of CpGs associated to the feature. Positive NES indicates hypermethylation and negative hypomethylation in tumors. Number of DMPs common presents the number of DMPs (fulfilling the criteria set for significant DMPs, see Methods) common in all tumor groups. DMRs common presents whether the gene is present in DMRs that are common for all LS tumor groups. Beta values vs. mRNA-expression presents the *R* value and BH-adjusted *P* value of the Spearman’s correlation test between *β* values and gene expression values by RNA sequencing of the overlapping LS samples (see Methods). Bolded promoter/gene indicate the negative correlation between *β* values and gene expression (i.e., hypermethylation and under-expression in tumors/hypomethylation and up-expression in tumors)

*Gene is common, but genomic region is different in adenomas vs. carcinomas

AdH, adenoma with high-grade dysplasia; AdL, adenoma with low-grade dysplasia; Ca, carcinoma; NES, normalized enrichment score by the number of CpGs associated to the feature

### Differentially methylated probes in FAP polyps

Compared to LS adenomas with low-grade dysplasia, the number of DMPs was much lower in FAP adenomas relative to their normal counterparts: 995 DMPs in FAP (26,848 in LS adenomas with low-grade dysplasia) (Fig. 5D; Additional Table 2). Also, the values of |Δ*β*| compared to normal counterparts were smaller in FAP adenomas: mean 0.117 (SD ± 0.017, range 0.10–0.24), whereas for LS adenomas with low-grade dysplasia, mean was 0.128 (SD ± 0.024, range 0.10–0.33) (t test two-sided *P* = 2.65e−69). To note, 44% (441/995) DMPs were found among common DMPs in all LS tumor groups (Additional Table 2; Additional Fig. 6), suggesting their general importance in colorectal tumorigenesis.

At probe-level, genes that were targeted with at least three probes and located in a CGI were *KAZALD1* (hypomethylation) and *RAB34* (hypermethylation). Genes targeted with two probes showing hypermethylation were *C9orf50*, *GATA2*, *ITGA4*, *LTBP4*, *NR5A2*, *MNX1*, and *OPLAH*, whereas the ones hypomethylated targeted *DIP2C*, and *MST1R* (Additional Table 2). Ten probes with the largest methylation difference are presented in Table 2B. The topmost probe was cg18973112 targeting *NTMT1*/*C9orf50*. In pathway analyses of DMPs in FAP adenomas, no GO (Additional Table 5 A), KEGG (Additional Table 5B), or GSA (Additional Table 5 C) results were retrieved with FDR < 0.05.

### Differentially methylated regions in FAP polyps

DMR analysis resulted in only two DMRs when we set |mean of Δ*β* (meandiff)|> 0.10 and FDR < 0.05 with overlapping genes of *FAM115A* and *B3GNT7*. When we lowered a cut-off of |meandiff|> 0.08, 22 DMRs were identified and of those, 18 were overlapping with genes (Additional Table 8). The regions with more than 10 CpGs were overlapping with *PITX1*, *VELF2*, *FBLIM1*, *OR2I1P*, *OPLAH*, and *TXNRD1*, *EID3*. Notably, of these largest DMRs, *FBLIM1*, *OR2I1P*, and *OPLAH* were identical or represented the same but somewhat smaller region than observed in all LS tumor groups (Additional Table 6), providing further support for a general role in CRC tumorigenesis.

In FAP tumors, when adjusting number of CpGs to three as a threshold used in the MCSEA promoter analysis, five promoters were hypermethylated: *RNH1* (Normalized Enrichment Score, NES, 2.5, BH-adjusted *P* value = 3.76e−05), *EREG* (NES 2.1, BH-adjusted *P* value = 5.11e−04), *RAB34* (NES 2.0, BH-adjusted *P* value = 7.04e−04), *C9orf50* (NES = 2.0, BH-adjusted *P* value = 7.04e−04), and *SERPINA1* (NES 1.6, BH-adjusted *P* value = 0.049). Moreover, *KAZALD1* was hypomethylated but with only suggestive values (NES −0.8, BH-adjusted *P* value = 0.72).

### Differentially methylated probes and regions in normal sample sets

The methylation changes were very subtle between LS normal and LS unaffected normal samples (Additional Table 2, Additional Table 8). Due to a very small number of DMPs, GO, KEGG or GSA analyses could not be utilized.

Differential methylation analysis revealed almost 7,824 DMPs between LS and FAP normal colon (Additional Table 2, Additional Fig. 5 C). If we set the |Δ*β*|> 0.20, still 916 DMPs remained significant, and 79 genes were represented with at least five probes. Ten probes with the largest methylation difference are presented in Table 2C. When we looked only at CGI- and 5′UTR/TSS200/TSS1500/1stExon-associated probes, 183 DMPs remained. There were three genes with multiple probes, all hypermethylated in FAP: *CDX2*, *GRHL3*, and *HOXA5*. Interestingly, 9% (663/7284) of DMPs were present among common DMPs in all LS tumor groups compared to normal LS mucosa, and 423/663 overlapped with genes (Additional Table 2, 3). All those DMPs that were hypermethylated in LS tumors were hypermethylated in FAP normal mucosa compared to LS normal mucosa, and vice versa, all that were hypomethylated in LS tumors were hypomethylated in FAP normal mucosa. Among the top findings, i.e., multiple probes targeting the same gene, and/or |Δ*β*|> 0.20, of common DMPs and differentially methylated promoters in all LS tumor groups (Table 4), the same probes (at least two) were altered similarly in FAP normal mucosa: *ARHGDIB*, *CNTN4*, *DAPP1*, *RIMS1, TCN1* (hypomethylated), and *FBLIM1*, *HOXA3*, *HOXA5*, *OPLAH*, *NR5A2*, *SEPT9, THBS1* (hypermethylated) (Additional Table 2, 3).

When pathway analyses were applied to DMPs between LS normal and FAP normal samples, alterations in inflammation-associated pathways of hypermethylated DMPs (in LS) constituted the most consistent finding (GSA analysis, Additional Table 5 A; Revigo analysis, Additional Table 5D).

DMR investigation revealed 367 DMRs, of which 272 overlapped with genes (Additional Table 8). The top ten DMRs overlapped with the following genes (some DMRs had more than one gene annotation, separated with comma): *HOXA*-*AS3*, *HOXA3*, *HOXA5*, *HOXA6*; *MEIS2*; *NR5A2*, *CELF2*; *KHDRBS2*; *HOXA2*; *PRAC2*, *PRAC1*, *MIR3185*, *HOXB13*; *SOGA2*; *TBX4*, and *HOXA-AS2*, *HOXA3*. They were mainly all hypermethylated in FAP normal, but regions overlapping with *CELF2* and *PRAC2*, *PRAC1*, *MIR3185*, *HOXB13* were hypermethylated in LS (Additional Table 8).

Promoter analysis resulted in 24 differentially methylated promoters between LS and FAP normal mucosa (Additional Table 7). The five promoters with the highest NES value included three different *HOXA* genes: *HOXA5*, *HOXA4,* and *HOXA3*, all hypermethylated in FAP. Promoters hypermethylated in FAP were in *HOXA5*, *HOXA3*, *ATP10A*, *SOX2*, *HOXA4*, *CDX2*, *MIR922*, *ADAMTSL1*, *GRHL3*, *KHDRBS2*, *FAM102A*, *CDYL*, *LITD1*, and *UGT1A6*. Hypermethylated in LS were *CUGBP2*, *GJB2*, *LOC100130872*-*SPON2*, *NEDD4L*, *FOXP1*, *THBS1*, *FILIP1*, *FRZB*, *GNG2*, and *RBM24*. Of those, *HOXA3* and *HOXA5* promoters were hypermethylated also in all LS tumor groups. In total, there were 24 promoters differentially methylated between LS and FAP normal mucosa, and 10 showed differential methylation also in at least one LS tumor (adenomas or carcinoma) compared to LS normal. Of those, eight (*HOXA3*, *HOXA5*, *ATP10A*, *SOX2*, *MIR922*, *ADAMTSL1*, *KHDRBS2*, and *LITD1*) were hypermethylated in at least one LS tumor group like here in FAP normal, whereas *NEDD4L*, and *FOXP1* were hypermethylated in LS carcinomas and here in LS normal (Additional Table 7). Intriguingly, *NEDD4L* methylation was negatively correlated with gene expression (Additional Table 6) and could serve as a potential field defect in normal colon mucosa for LS-associated cancer.

### Common versus unique promoter alterations in all comparisons

We focused on the promoter analysis results as they provide deeper insight into the large-scale methylation alterations and are likely to be associated with gene expression. No promoter was shared in all comparisons. In search for differentially methylated promoters unique to a single comparative setting, hypermethylation of *EREG* was found unique to FAP adenomas. Of the hypermethylated promoters in FAP normal (vs. LS normal), *CDX2*, and *UGT1A6* were unique to FAP normal. *LOC100130872*-*SPON2*, *FRZB*, and *RBM24* were hypermethylated in LS normal only. Promoters that were differentially methylated in all LS tumor groups but not in FAP adenoma vs. FAP normal or LS normal vs. FAP normal comparisons were *ADHFE1*, *EYA4*, *FLI1*, *GRIA4*, *KLF17*, *LBX2*, *PREX2*, *RIMS1*, and *ZNF528*.

## Discussion

In this study, we utilized genome-wide EPIC methylation array to investigate DNA methylation alterations in colorectal tumors arising on two different hereditary backgrounds: LS and FAP. Differential methylation was investigated at CpG site (probes) and regional level. While differences were observed, the overall genome-wide differential methylation in various genomic regions related to CGIs occurred very similarly in LS and FAP tumors compared to their normal counterparts (Fig. 4). Most of the DMPs in open sea regions were generally hypomethylated in all tumors, but in LS carcinomas, more than 30% of the DMPs targeting open sea regions were hypermethylated and CGI hypermethylation proportionally decreased compared to LS or FAP-associated adenomas (Fig. 4B). This can suggest the importance of CGI hypermethylation as an early tumorigenic event. Recently, Ibrahim et al. investigated DNA methylation events in multiple different cancers, including CRC, and reported that most DMPs were in open sea regions, followed by CGI and shores and shelves [58]. In our data, the difference between CGIs and shores and shelves was not as evident. Related to gene regions, Ibrahim et al. reported that most DMPs were in gene bodies followed by intergenic regions, TSS1500, TSS200, 5′UTR, 3′UTR, and 1 st exon. Generally, like in our study, either intergenic or gene body regions are most often altered followed by other gene-related regions, and of CGI-associated regions, primarily open sea is affected in colorectal tumors compared to normal colon [59–61].

In LS tumors, hypermethylation increased with the grade of dysplasia in all gene regions (Fig. 4C), and numbers of DMPs, DMRs and differentially methylated promoters were the most abundant in LS adenomas with high-grade dysplasia (Results; Fig. 4A, C; Fig. 5A–C; Additional Fig. 6 A). Recent studies on sporadic colon adenomas and cancer have reported similar findings: there were more DMPs in adenomas than in cancer, when compared to normal adjacent colon, although the proportion of hypermethylated probes increased in cancer [60, 61]. Of all DMPs, a vast majority were hypomethylated, which may be explained by a large proportion of probes targeting intergenic regions on the Illumina array and those regions being prone to hypomethylation in cancer, as discussed by Ibrahim et al. [58]. Proportionally, intergenic regions were overrepresented as DMP sites in FAP adenomas compared to LS tumors (Fig. 4C). Hypomethylated regions in open sea regions and shores of the CGIs occurring also in premalignant solid tumors, have been associated with CIN [62, 63], but in this study, no clear difference was observed between LS and FAP tumors, although latter ones are usually characterized by CIN [8, 21]. Interestingly, LINE-1 hypomethylation that is linked to CIN was more marked in LS adenomas than in FAP adenomas (Fig. 2A). This is in line with our recent data emphasizing the important and early role of CIN-related mitotic abnormalities in colorectal tumorigenesis in LS [51].

We used Horvath’s methylation clock to assess the DNAm age in colorectal samples. Recently, it was validated as most accurate tool for estimating the chronological age in colon normal mucosa samples [64]. DNAm age analysis showed different DNAm aging in LS and FAP normal samples and indicated a difference between proximal and distal colon (Fig. 3, Additional Fig. 1). Cuadros et al. [27] observed high variability in DNAm age of LS patients based on peripheral blood samples. The high variability was also seen in this study, especially in LS normal samples (Fig. 3), and in proximal colon (Additional Fig. 1). Accelerated DNAm age was reported in FAP normal colon organoids compared to healthy subjects [28]. Our FAP samples (both normal and adenomas) displayed DNAm age acceleration compared to LS samples (Fig. 3A), although statistically non-significant. To the best of our knowledge, studies by Cuadros et al. [27] and Devall et al. [28] are the only previous ones investigating DNAm age in LS and FAP, respectively.

LS and FAP tumors were compared to their normal counterparts to find plausible colorectal tumorigenesis-associated methylation alterations occurring already in adenomas. DNA methylation alterations were 30–50-fold more prevalent in LS adenomas than in FAP adenomas (Fig. 5, Additional Fig. 6), and the changes were abundant already in LS adenomas with low-grade dysplasia and even before MSI (Fig. 2C–D). This underscores the DNA methylation alterations occurring very early in the colorectal tumorigenesis, as many studies on sporadic colon adenomas with genome-wide methylation approach have proven [60, 61, 65, 66]. There is extensive evidence of DNA methylation alterations as field defects in normal colon mucosa [66–77]. Our study suggests that FAP normal mucosa is more prone to acquire those CRC-associated methylation alterations, as 45% of all DMPs in FAP normal colon (when compared to LS normal colon) were similarly altered in at least one group of LS tumors, and 10% were altered in all LS tumor groups, always alike hyper- or hypomethylated (Additional Fig. 6; Additional Tables 2, 3). This may indicate certain CRC tumorigenesis-associated DNA methylation alterations to occur as field defects earlier in *APC*-driven tumorigenesis. *APC*-driven predisposition increases cancer initiation, and one mutated allele is sufficient to initiate the tumorigenesis [78], and this is where altered DNA methylation targeting various growth-related genes may be one of the early mechanisms accelerating the tumorigenic process. On the other hand, LS tumors display a vast abundance of DNA methylation alterations, which may in part reflect hypermutability due to MMR defect, with epigenetic regulatory genes as targets [30]. Indeed, our data confirmed that increased TMB correlated positively with increased hypermethylation [79, 80], and global hypomethylation (LINE-1 methylation as a surrogate) (Fig. 2F, G) [81–83]. Regarding the plausible field cancerization, exact information of distance to the tumor was not included in our sampling data. However, previous studies have given evidence that field cancerization may occur within up to 10 cm distance from the tumor [72, 84].

In a recent study on normal colon organoids derived from FAP and healthy subjects, *KAZALD1* hypomethylation and its increased gene expression in FAP-associated organoids was one of the most important findings [28]. Also in our study, four DMPs overlapping with *KAZALD1* were hypomethylated (Additional Table 2). In our study, LS adenomas with low- and high-grade dysplasia also displayed hypomethylated *KAZALD1* DMPs in promoter area (*n* = 2 and 4, respectively) (Additional Table 2).

Our pathway analysis of hyper- and hypomethylated DMPs on promoter regions suggested that functional targets for hyper- and hypomethylation are different. Pathway analysis underscores that in LS tumors, especially in LS carcinomas, inflammation-related pathways are targeted by hypomethylation (Table 3; Additional Table 5A–D). This agrees with the high immunogenicity of LS tumors due to the accumulation of novel frameshift peptides caused by MMR defect [85]. On the other hand, hypermethylation was associated with epithelial to mesenchymal transition as evident in LS adenomas (Additional Table 5 C), and olfactory transduction similar to that reported previously in colorectal adenomas [60].

Our LS sample material was partially overlapping with the material used in our recent study [51], and it allowed us to compare methylation data with transcriptome data. Differentially methylated promoters and regions identified in LS tumors were compared with gene expression and several confirmed to display negative correlation. Of 22 differentially methylated promoters common between all LS tumor groups, seven inversely correlated with expression: *ADHFE1*, *DAPP1*, *FBLIM1*, *GRIA4*, *HOXA3*, *KLF17*, and *ZNF528* (Table 4). In total, 41 DMRs involving the same overlapping genes and that inversely correlated with gene expression were common in all LS tumor groups. The topmost findings with the strongest negative correlation were: *DAPP1*, *PITPNC1*, *NR5A2*, *RP11-466L17.1*, *MET*, *TCN1*, *MYT1L*, *ITGBL1*, *ARHGDIB*, and *AFF1*. These changes occurred already in adenomas with low-grade dysplasia suggesting their functional role in early stages of tumorigenesis. Numerous of these alterations have previously been reported in colon adenomas and/or carcinomas as topmost findings [59–61, 64, 65, 86–90]. In our study, hypermethylation of homeobox (HOX) genes stood out in all LS tumor groups and in FAP normal colon. Their role in cancer is widely known and differential methylation has been reported even in aberrant crypt foci of colon [91].

Of differentially methylated promoters shared by all LS tumor groups (Table 4), nine promoters (*ADHFE1*, *EYA4*, *FLI1*, *GRIA4*, *KLF17*, *LBX2*, *PREX2*, *RIMS1*, and *ZNF528*) were altered only in LS tumors, but many have been associated with sporadic CRC and may represent early changes [60, 76, 86, 92–94]. LS normal colon mucosa displayed hypermethylation of *FOXP1* and *NEDD4L* as plausible tumorigenic DNA methylation alterations (Additional Table 2). Promoter hypermethylation of those genes was observed in LS normal mucosa (compared to FAP) and LS carcinoma, and methylation of *NEDD4L* was inversely correlated with gene expression. *FOXP1 *[95] and *NEDD4L* [96] are plausible tumor suppressor genes in colon cancer. Hypermethylation of *CDX2* (as well as *UGT1A6*) was seen only in FAP normal colon (when compared to LS normal colon) (Additional Table 2). *CDX2* is a recognized tumor suppressor gene and its hypermethylation is proposed as an adverse prognostic biomarker in CRC and is linked to the serrated pathway [97, 98]. *LOC100130872*-*SPON2*, *FRZB (SFRP3)*, and *RBM24* were only hypermethylated in LS normal (compared to FAP normal) (Additional Table 2). The protein products of *FRZB* [99, 100] and *RBM24 *[101] have tumor suppressive roles in colorectal and other cancers. Hypermethylation of *EREG* promoter was unique to FAP adenomas (compared to FAP normal) (Additional Table 2). Other studies have found that *EREG* is generally upregulated in cancer [102] which may result from CGI hypomethylation [61, 103].

Of the topmost findings based on the largest Δ*β* values (Table 2), *SND1* (cg09296001) was identified in all LS tumor groups and FAP adenomas. It has previously been linked to sporadic CRC [60, 61, 86, 104] and is proposed as a biomarker of CRC [60]. Also, hypermethylation of *OPLAH* in CRC is a recurrent finding and often caught even with the same probes [59, 61, 86, 92]. *B3GNTL1* cg10344477 has been found among highly methylated probes in CRC [105], but in our study, the probe was hypomethylated in LS and FAP tumors (Table 2).

Taken together, the possibility to compare LS- and FAP-associated colorectal samples, normal and neoplastic, for DNA methylation alterations genome-wide is a major advantage of this investigation and expands the existing limited knowledge. In LS, the availability of RNA-sequencing data additionally allowed us to explore expressional consequences of altered DNA methylation. Our finding of LS- and FAP-associated methylation changes resembling those recurrently reported from sporadic colon tumors is likely to prompt future comparative investigations to shed light on early epigenetic changes in colorectal tumorigenesis against different states of predisposition. The fact that we lacked information of the exact distance between tumor samples and adjacent normal mucosae is a possible drawback that needs to be considered in interpretations of our results. Moreover, our study did not allow assessment of methylation alterations in relation to the mutated MMR gene, as *MLH1* mutation carriers were overrepresented in this series (Table 1). There is growing evidence that the predisposing MMR gene may significantly influence LS-associated colorectal tumorigenesis [11, 106], and the possible dependence of DNA methylation changes and their timing on the constitutionally mutant MMR gene remains to be addressed by future studies.

## Conclusions

In conclusion, multiple CRC-associated DNA methylation changes distinguished FAP normal colon mucosa paired to tumor, suggesting that DNA methylation may play a crucial role in field cancerization in *APC*-driven tumorigenesis. In LS adenomas, the number of methylation alterations was approximately 30–50-fold higher than in FAP adenomas when compared to their normal counterparts. These findings together highlight the importance of DNA methylation alterations at early stages of colorectal tumors and even in normal colon mucosa, which, apart from elucidating tumorigenic mechanisms, has clinical relevance. Our observation that many methylation changes present in hereditary colorectal tumors may be shared with sporadic CRC suggests that they could serve as biomarkers for diagnosis and prognosis of CRC irrespective of genetic background. Cancer-associated methylation markers can be detectable in cell-free DNA [107, 108] before the diagnosis [109], and especially if used in a multi-omics or multi-marker approach [110, 111], could offer a noninvasive tool for CRC detection, and thereby supplement regular colonoscopy surveillance in individuals with increased risk of CRC. To design specific tests, it is crucial to understand the timing and significance of tumorigenic changes in CRC with or without hereditary predisposition.

## Supplementary Information

Additional file 1.

Additional file 2.

Additional file 3.

Additional file 4.

Additional file 5.

Additional file 6.

Additional file 7.

Additional file 8.

Additional file 9.

Additional file 10.

Additional file 11.

Additional file 12.

Additional file 13.

## Data Availability

The datasets generated and analyzed during the current study are not publicly available because our IRB approvals do not allow sharing raw genomic data from the patients but are available from the corresponding author on reasonable request.

## Abbreviations

BH: Benjamini-Hochberg
BP: Biological process
CGI: CpG island
CIMP: CpG island methylator phenotype
CIN: Chromosomal instability
CRC: Colorectal cancer
DMP: Differentially methylated probe
DMR: Differentially methylated region
DNAm: DNA methylation
FAP: Familial adenomatous polyposis
FDR: False discovery rate
GO: Gene Ontology
GSA: Gene set analysis
KEGG: Kyoto Encyclopedia of Genes and Genomes
LINE-1: Long interspersed element 1
LS: Lynch syndrome
Mb: Mega base
MMR: Mismatch repair
MSI: Microsatellite instable
MSS: Microsatellite stable
NES: Normalized enrichment score
ns: Non-significant
N_Shore/N_Shelf: The CpG shore/shelf located 5′ to the island
S_Shore/S_Shelf: The CpG shore/shelf located 3′ to the island
TMB: Tumor mutation burden
TSS: Transcription starting site
