# Supplementary material for: Genome-wide DNA methylation profiles of colorectal tumors in Lynch syndrome and familial adenomatous polyposis
Source: Clin Epigenetics. 2025 Aug 2;17:137. doi: 10.1186/s13148-025-01940-x (PMC12317532; doi:10.1186/s13148-025-01940-x)
Supplement: Supplementary file 4 — Additional file 4. [file 13148_2025_1940_MOESM4_ESM.docx]

**Additional Table 4**. **Numerical summary of GO, KEGG and GSA analysis results**. Table presents the number of gene ontologies for biological processes (GO BP), KEGG pathways and gene sets (GSA) with adjusted *P* value < 0.05 (unless indicated with an asterisk, see the footnote), when either all resulted DMPs, hypo- or hypermethylated ones were used as an input. Tumor sample groups were compared to normal counterparts.

| **Sample group** | **DMPs** | **GO BP** | **Revigo Reduced BP (unique parentTerm)** | **KEGG** | **GSA** |
| --- | --- | --- | --- | --- | --- |
| **LS adenoma_LG** | **all** | 153 | 28 | 5 | 2 |
|  | **hypo** | 118 | 23 | 6 | 2 |
|  | **hyper** | 28 | 6 | 3 | 0 |
| **LS adenoma_HG** | **all** | 292 | 35 | 14 | 4 |
|  | **hypo** | 109 | 23 | 7 | 4 |
|  | **hyper** | 467 | 48 | 13 | 3 |
| **LS carcinoma** | **all** | 118 | 22 | 3 | 4 |
|  | **hypo** | 235 | 26 | 10 | 3 |
|  | **hyper** | 1 (152)* | 0 (31)* | 0 (1)* | 5 |
| **FAP adenoma*** | **all** | 132 | 35 | 3 | 1 |
|  | **hypo** | 49 | 19 | 6 | 2 |
|  | **hyper** | 265 | 38 | 1 | 0 |
| **LS normal vs. FAP normal*** | **All** | 164 | 35 | 6 | 4 |
|  | **Hypo** | 75 | 19 | 3 | 2 |
|  | **hyper** | 200 | 35 | 3 | 7 |

* unadjusted *P* value < 0.01 used as a cut-off ((nearly) all FDR values > 0.05); LS carcinoma hypermethylated probes-based results are indicated in brackets with unadjusted p value < 0.01 (only one GO with FDR < 0.05)
