# Supplementary material for: Genome-wide DNA methylation profiles of colorectal tumors in Lynch syndrome and familial adenomatous polyposis
Source: Clin Epigenetics. 2025 Aug 2;17:137. doi: 10.1186/s13148-025-01940-x (PMC12317532; doi:10.1186/s13148-025-01940-x)
Supplement: Supplementary file 12 — Additional file 12. [file 13148_2025_1940_MOESM12_ESM.docx]

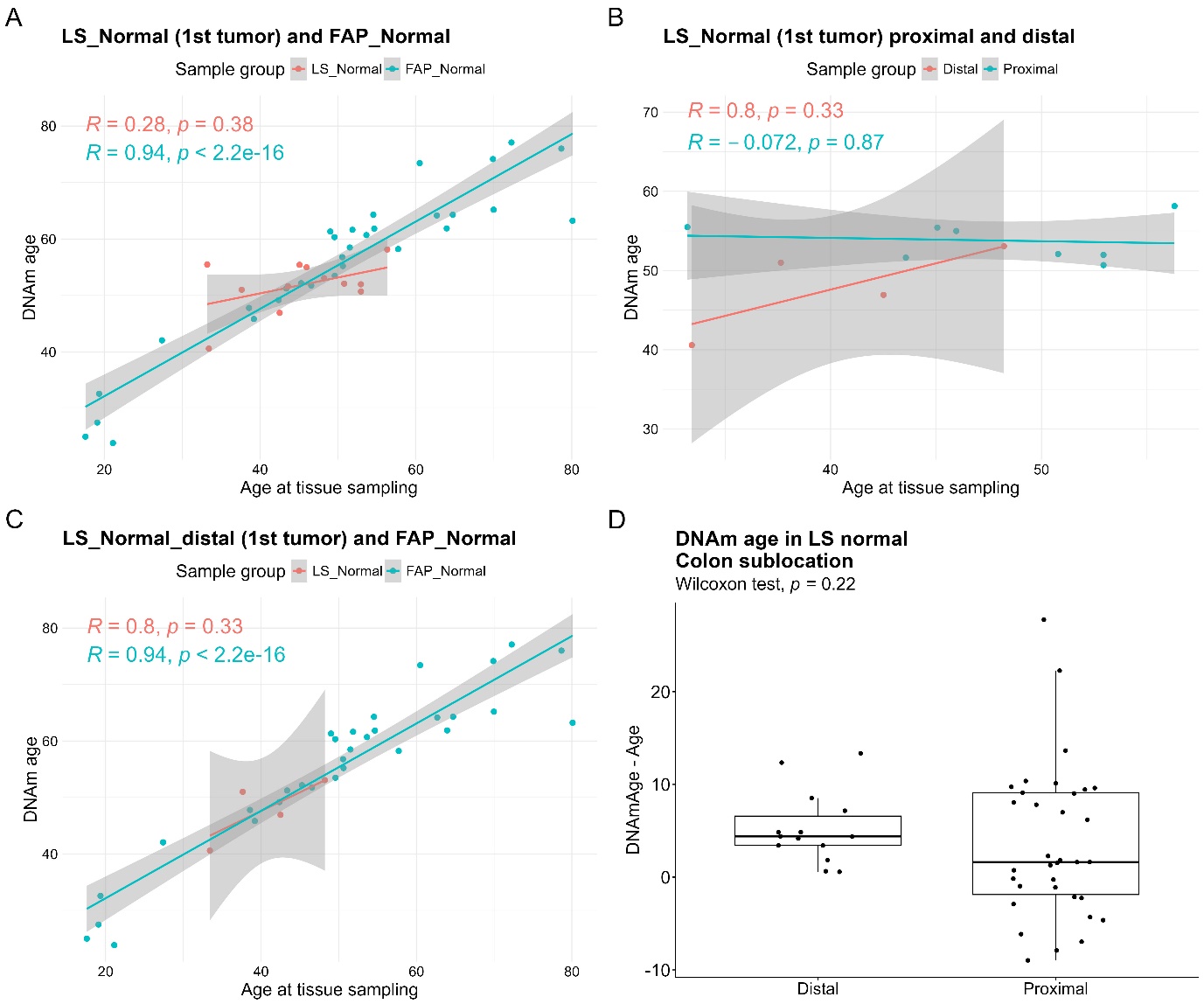


**Additional Figure 1. DNAm age versus age at the time of tissue sampling in normal colon samples.** **A**. LS normal paired to patients’ first tumor diagnosis and FAP normal samples. **B**. LS normal samples divided into proximal and distal samples. **C**. Distal samples of LS normal and FAP normal. The shaded areas represent the 95% confidence intervals. Correlations are calculated using the Spearman’s test. **D**. The difference of DNA methylation age and age at the sampling of normal mucosa from tumor-affected and unaffected LS carriers compared with colon sublocation. In the boxplot, the upper and lower edges of the boxes indicate the 75th and 25th percentiles, the horizontal line inside the box denotes the median, and the whiskers indicate the lowest and highest values (outliers are shown outside the whiskers). Each samples’ averaged *β*-value is indicated as a single dot.

**Additional Figure 2. Multidimensional scaling (MDS) plot of 1000 most variable CpGs.** A MDS plot illustrating the top 1000 most variable CpGs based on normalized and filtered *β*-values. Samples are colored by sample group (histology).


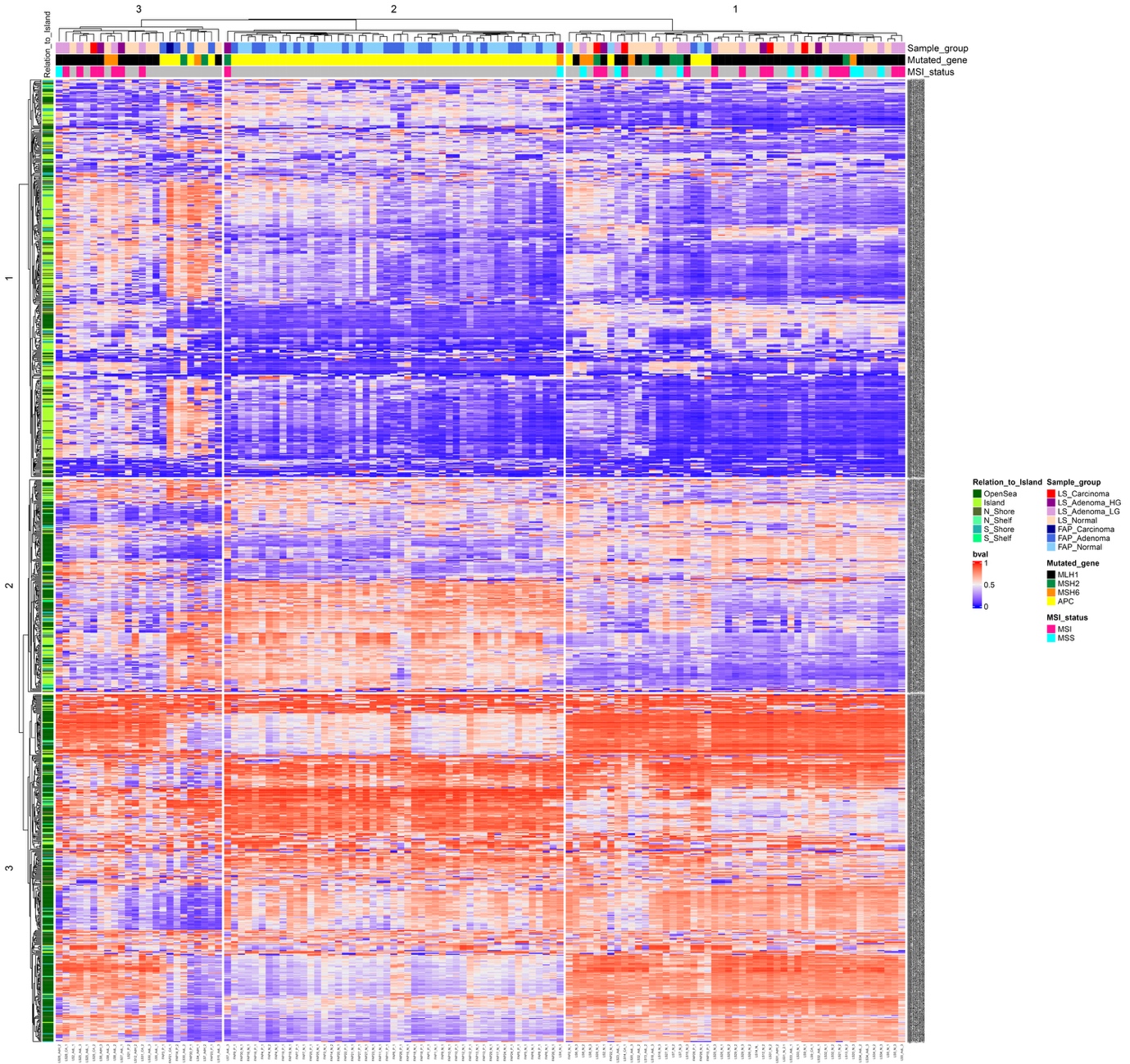


**Additional Figure 3. Heatmap of 1000 most variable CpGs**. A heatmap illustrating the top 1000 most variable CpGs based on normalized and filtered *β*-values. Sample group, mutated gene and MSI-status are annotated to the top of the heatmap. Relation to island is annotated to the left of the heatmap. Please note, this figure is also available as a PDF.


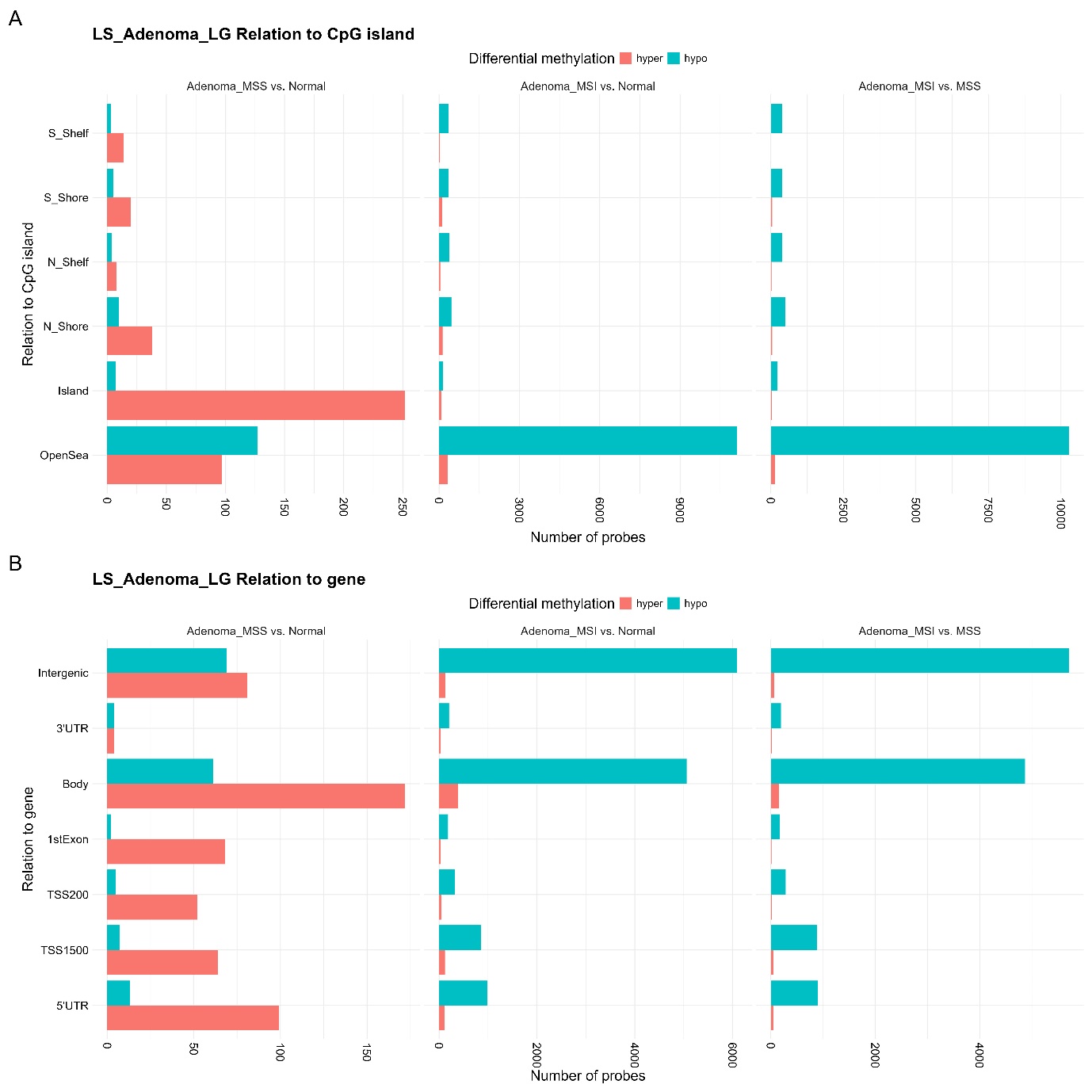


**Additional Figure 4. Overview of differential methylation in LS adenomas with low-grade dysplasia divided into MSS and MSI samples.** Red color indicates hypermethylated probes and blue stands for hypomethylated probes. **A**. DMPs in MSS and MSI adenomas when compared to LS normal, and DMPs between MSS and MSI adenomas, and their relation to CpG island (see Results for cut-off values). **B**. DMPs and their relation to gene regions.


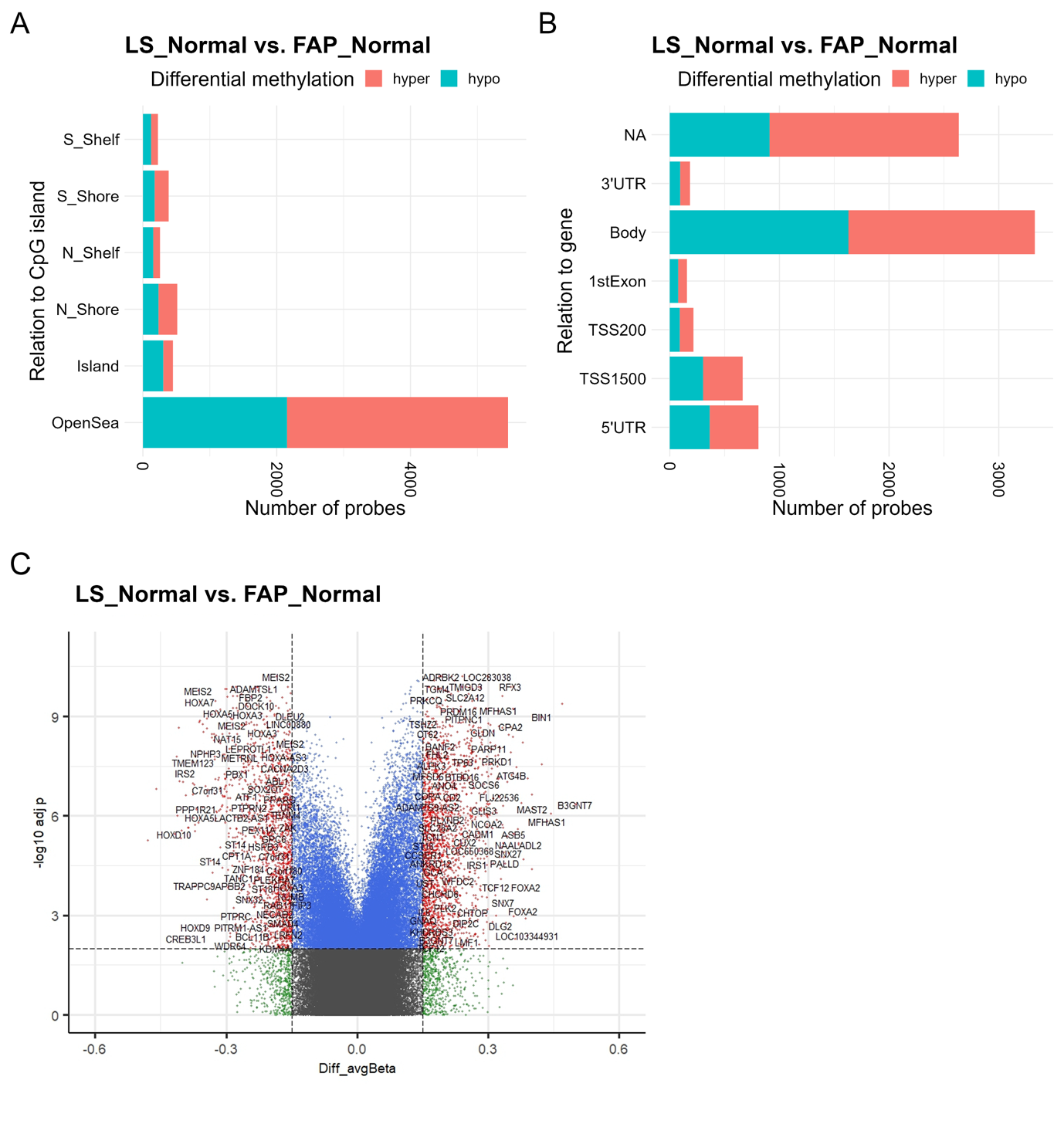


**Additional Figure 5.** **An overview of DMPs between LS normal and FAP normal colon and relation to CGI and gene regions**. LS normal and FAP normal sample groups were compared and methylation alterations (hypomethylation or hypermethylation) in LS normal were expressed relative to methylation levels in FAP normal. Red color indicates hypermethylated probes (in LS) and blue stands for hypomethylated probes (in LS). **A**. Overview of DMPs and their relation to CpG island. **B.** Overview of DMPs and their relation to gene regions. **C**. Volcano plot of DMPs. Adjusted *P* values are plotted against averaged *β*-values (Diff_avgBeta). Hypermethylated probes in tumors compared to normal counterparts are represented with positive averaged *β*-values and hypomethylated probes with negative values. Red dots indicate the DMPs above both thresholds marked with dashed lines (|Δ averaged *β*| > 0.15 and BH-adjusted -Log_10_ *P* > 2, i.e. *P* value < 0.01).


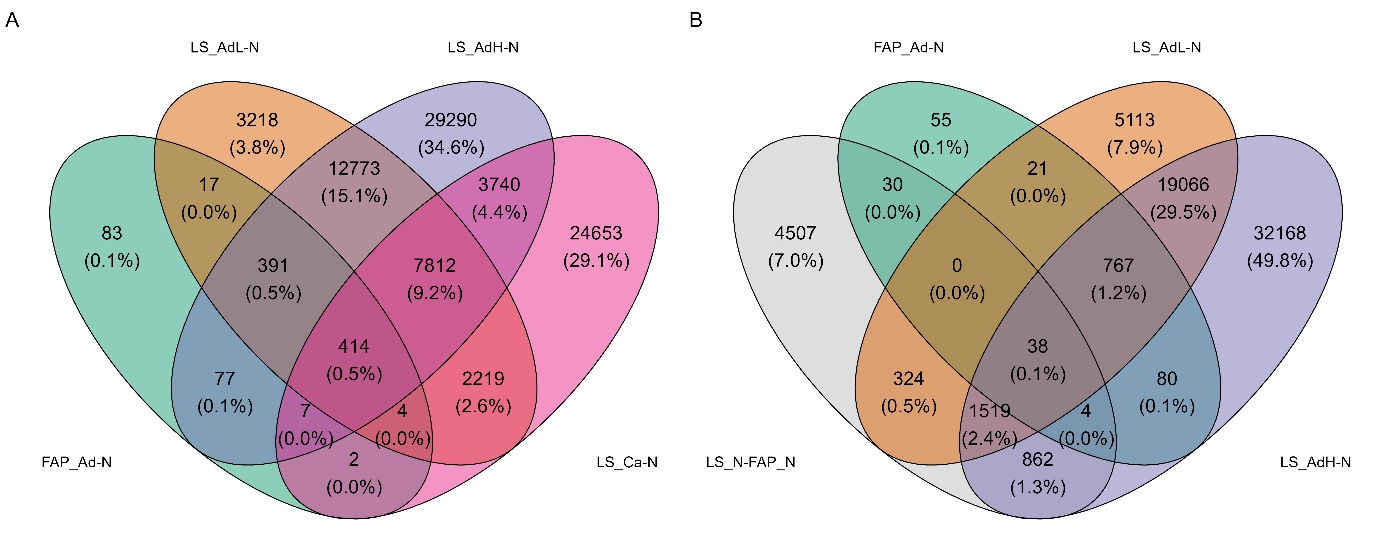


**Additional Figure 6. DMPs across FAP and LS samples. A**. DMPs in FAP adenomas and LS tumors compared to normal counterparts. **B**. DMPs in LS normal compared to FAP normal, and in LS tumors compared to normal counterparts. Percentages are calculated from a total number of all unique DMPs resulted from at least one of the comparisons in question. Abbreviations: FAP_Ad, FAP adenoma; FAP_N, FAP normal; LS_AdL, LS adenoma with low-grade dysplasia; LS_AdH, LS adenoma with high-grade dysplasia; LS_Ca, LS carcinoma, N, normal counterpart.
