# Supplementary figures and images for: Genome-wide DNA methylation profiles of colorectal tumors in Lynch syndrome and familial adenomatous polyposis
Source: Clin Epigenetics. 2025 Aug 2;17:137. doi: 10.1186/s13148-025-01940-x (PMC12317532; doi:10.1186/s13148-025-01940-x)

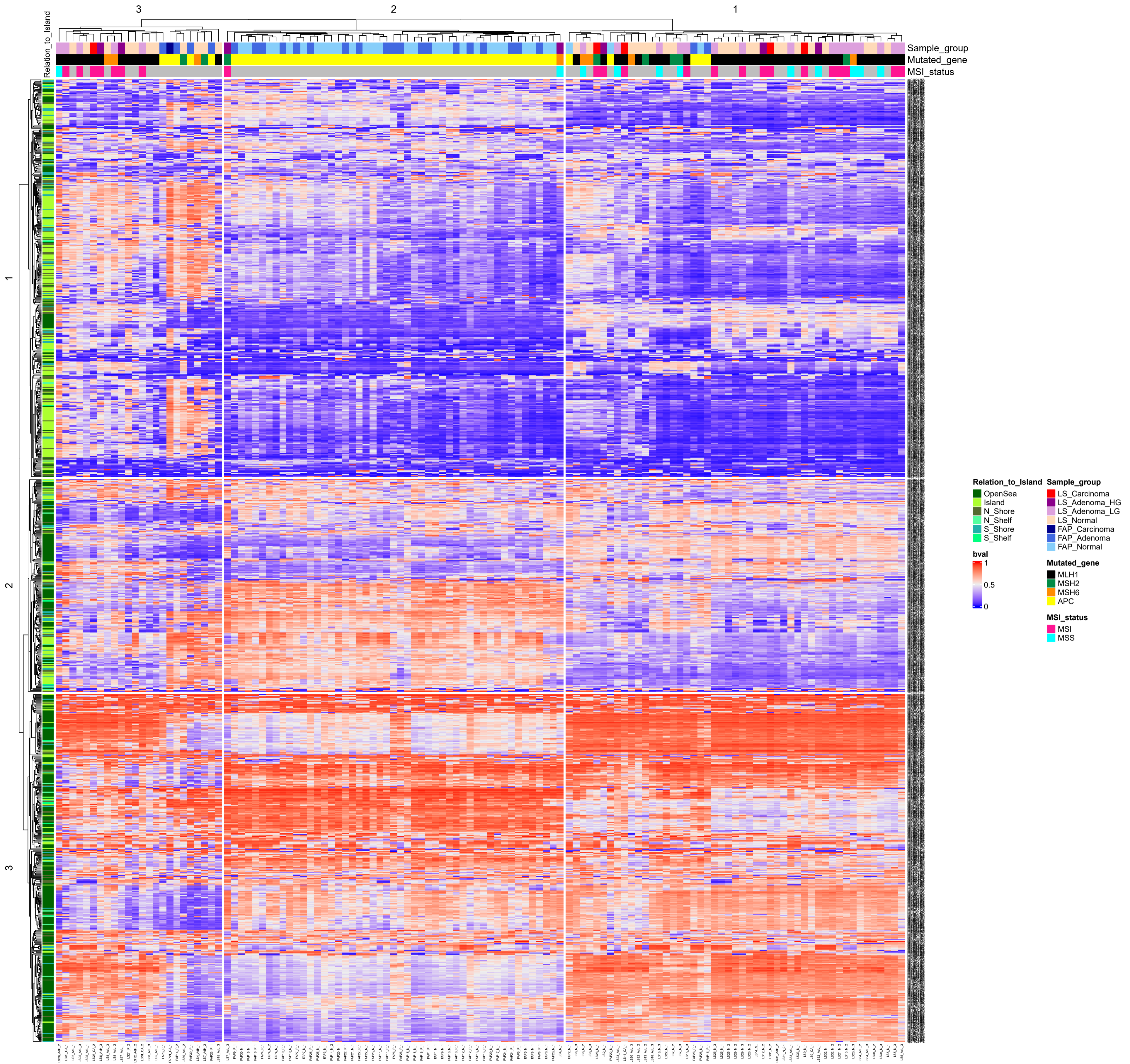

Supplement: Supplementary file 13 — Additional file 13. [file 13148_2025_1940_MOESM13_ESM.pdf]
